# Biomarkers of Extracellular Matrix Remodelling Are Linked to Severity and Outcome of Advanced Chronic Liver Disease

**DOI:** 10.1111/apt.70407

**Published:** 2025-10-12

**Authors:** Benedikt Simbrunner, Ida Falk Villesen, Georg Semmler, Lorenz Balcar, Georg Kramer, Joana Almeida Calvão, Benedikt Silvester Hofer, Mathias Jachs, Lukas Hartl, Jurij Maurer, Bernhard Scheiner, Kerstin Zinober, Rodrig Marculescu, Michael Trauner, Morten Karsdal, Thomas Reiberger, Mattias Mandorfer, Diana Julie Leeming

**Affiliations:** ^1^ Division of Gastroenterology and Hepatology, Department of Medicine III Medical University of Vienna Vienna Austria; ^2^ Vienna Hepatic Hemodynamic Lab, Division of Gastroenterology and Hepatology, Department of Medicine III Medical University of Vienna Vienna Austria; ^3^ Clinical Research Group MOTION Medical University of Vienna Vienna Austria; ^4^ Christian Doppler Laboratory for Portal Hypertension and Liver Fibrosis Medical University of Vienna Vienna Austria; ^5^ CeMM Research Center for Molecular Medicine of the Austrian Academy of Sciences Vienna Austria; ^6^ Center for Liver Research, Department of Gastroenterology and Hepatology Odense University Hospital Odense Denmark; ^7^ Department for Clinical Research University of Southern Denmark Odense Denmark; ^8^ Hepatology Unit, Internal Medicine Department Unidade Local de Saúde de Trás‐os‐Montes e Alto Douro (ULSTMAD) Vila Real Portugal; ^9^ Klinik Landstrasse 4. Medizinische Abteilung für Gastroenterologie und Hepatologie Vienna Austria; ^10^ Department of Laboratory Medicine Medical University of Vienna Vienna Austria; ^11^ Nordic Bioscience Herlev Denmark

**Keywords:** biomarkers, cirrhosis, extracellular matrix, HSC, portal hypertension

## Abstract

**Background:**

Extracellular matrix (ECM) remodelling in advanced chronic liver disease (ACLD) is characterised by hepatic fibrosis and (sinusoidal) basement membrane development contributing to portal hypertension (PH) and clinical complications.

**Methods:**

Patients with stable ACLD (*n* = 232) undergoing hepatic venous pressure gradient (HVPG) measurement were included. Blood biomarkers reflecting fibrosis formation (PRO‐C3, PRO‐C6, PRO‐C4 and PRO‐C18L) and degradation (C3M, C4M and C6Ma3), as well as the Enhanced Liver Fibrosis (ELF) score were measured. Associations with disease severity and liver‐related events (LRE: decompensation, acute‐on‐chronic liver failure, or liver‐related death) were analysed.

**Results:**

The cohort included 131 (57%) patients with decompensated ACLD (dACLD), median HVPG 18 (13–21) mmHg and MELD 11 (9–14). ECM remodelling biomarkers increased in patients with dACLD and with PH severity (HVPG 6–9: *n* = 28, 10–19: *n* = 124, ≥ 20 mmHg: *n* = 80; all *p* < 0.05), except PRO‐C18L (*p* = 0.231). Collagen degradation markers C3M and C4M—but not C6Ma3—also increased with HVPG (*p* < 0.01). Median follow‐up was 28.9 (IQR 12.0–43.6) months. Fibrogenesis biomarkers (ELF, PRO‐C3/‐C4/‐C6) were predictive of first decompensation in cACLD, while no clinically meaningful association with LRE was observed in dACLD. In multivariate Cox regression models adjusted for HVPG, MELD, albumin, and decompensation state, PRO‐C4 (aHR per 100 ng/mL: 1.22, *p* < 0.001), C3M (aHR per ng/mL: 0.98, *p* = 0.044), and C4M (aHR per ng/mL: 0.98, *p* = 0.084) displayed independent prognostic value for LRE. The predictive value of fibrogenesis biomarkers for first decompensation (ELF, PRO‐C3, PRO‐C6) was validated in an independent cACLD cohort (*n* = 185).

**Conclusion:**

ECM remodelling biomarkers reflect PH and disease severity in ACLD. Their prognostic value for disease progression is largely restricted to cACLD. Future studies should investigate whether repeated measurements improve risk stratification.

**Trial Registration:** NCT03267615

## Graphical abstract

Extracellular matrix (ECM) remodelling biomarkers reflect portal hypertension and disease severity in advanced chronic liver disease (ACLD). However, their predictive value for disease progression is largely restricted to compensated ACLD (cACLD).

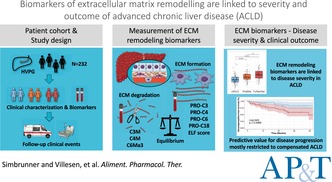

**Abbreviations**
ACLF: acute‐on‐chronic liver failure
AD: acute decompensation
ALD: alcohol‐related liver disease
AUROC: area under the receiver operating characteristic curve
c/dACLD: compensated/decompensated advanced chronic liver disease
C3M: collagen type III degradation biomarker
C4M: collagen type IV degradation biomarker
C6Ma3: collagen type VI degradation biomarker
CRP: C‐reactive protein
CSPH: clinically significant portal hypertension
EASL: European Association for the Study of the Liver
ECM: extracellular matrix
ELF: Enhanced Liver Fibrosis
HCC: hepatocellular carcinoma
HCV: hepatitis C virus
HE: hepatic encephalopathy
HRS‐AKI: hepatorenal syndrome acute kidney injury
HSC: hepatic stellate cells
HVPG: hepatic venous pressure gradient
IL‐6: interleukin‐6
IQR: interquartile range
LBP: lipopolysaccharide‐binding protein
LRD: liver‐related death
LSM: liver stiffness measurement
MELD: Model for End‐Stage Liver Disease
MetALD: metabolic dysfunction and alcohol‐related liver disease
NIT: noninvasive test
NSBB: nonselective beta‐blockers
PH: portal hypertension
PIIINP: procollagen III N‐terminal peptide
PRO‐C18L: collagen type XVIII formation biomarker
PRO‐C3: collagen type III formation biomarker
PRO‐C4: collagen type IV formation biomarker
PRO‐C6: collagen type VI formation biomarker
SBP: spontaneous bacterial peritonitis
TIMP‐1: tissue inhibitor of metalloproteinases‐1

## 1 Introduction

Advanced liver fibrosis is a key pathological feature of advanced chronic liver disease (ACLD). Fibrosis arises from continuous liver injury that triggers hepatocyte death, chronic inflammation and the activation of hepatic stellate cells (HSCs). Activated HSCs produce extracellular matrix (ECM) components, leading to the accumulation of collagens and other ECM molecules [1]. Progression of fibrosis causes distortion of the liver architecture, impairing its function and ultimately contributing to the development of cirrhosis and its complications, such as portal hypertension (PH), hepatic decompensation and liver failure [2].

The assessment of fibrosis in ACLD is crucial for determining disease severity, guiding therapeutic management and prognostication. Quantification of fibrosis on liver biopsy samples, as reflected by collagen staining, has been shown to predict prognosis within different liver disease etiologies [3, 4, 5]; however, due to the emergence of noninvasive methods and its invasiveness, liver biopsy is rarely performed for prognostic purposes nowadays in clinical routine. Serological biomarkers reflecting ECM remodelling are gaining recognition as a noninvasive alternative for disease staging and risk stratifying patients with chronic liver disease [6]. For example, the Enhanced Liver Fibrosis (ELF) score incorporating serum levels of hyaluronic acid, tissue inhibitor of metalloproteinases‐1 (TIMP‐1), and procollagen III N‐terminal peptide (PIIINP) reflects different components of hepatic ECM remodelling [7, 8]. Similarly, the ECM‐related biomarkers PRO‐C3, PRO‐C4 and PRO‐C6, which represent the formation of type III, IV and VI collagen, respectively, and their degradation products (C3M, C4M and C6Ma3), were designed to reflect the dynamic processes of fibrogenesis (the formation of ECM) and fibrolysis (the degradation of ECM) [6]. In addition to the discrimination of fibrosis stages based on histology [9, 10, 11, 12, 13], these biomarkers have been shown to correlate with essential disease‐driving mechanisms in ACLD such as PH and systemic inflammation [14, 15], as well as disease regression, and have proven useful for prediction of clinical events in patients with chronic liver disease [8, 16].

In the context of ACLD, the balance between the ECM biomarkers reflecting fibrosis remodelling, specifically ECM formation and degradation, and their relationship with disease severity and prognosis remains poorly understood. This study investigated the association between ECM remodelling biomarkers of the interstitial matrix, basement membrane, and clinical stage, PH, and prognosis in a prospective cohort of ACLD patients undergoing hepatic venous pressure gradient (HVPG) measurement.

## 2 Patients and Methods

### 2.1 Study Design and Clinical Characterisation

Patients with ACLD (confirmed by an HVPG ≥ 6 mmHg) undergoing liver vein catheterization were prospectively recruited into the Vienna Cirrhosis Study (VICIS) between January 2017 and June 2020, at the Medical University of Vienna, Austria. Patients with posthepatic or presinusoidal PH, previous transjugular intrahepatic portosystemic shunt or liver transplantation, hepatocellular carcinoma beyond Milan criteria, other active malignancies, and treatment with nonselective beta‐blockers, bacterial infection, or nonelective hospitalisation at the time of HVPG measurement were excluded by review of prospectively collected data (Figure S1). Notably, the present cohort and associated clinical and laboratory data overlap with previous publications that analysed the predictive value of ELF for clinically significant PH [17], and the relationship between ECM remodelling and systemic inflammation, i.e., focusing on different clinical and pathophysiological aspects [14]. Hepatic decompensation events previous to the time point of HVPG measurement were determined according to national and European guidelines [18, 19] and Baveno VII consensus criteria [20]. The validation cohort was recruited at HVPG measurement between July 2020 and August 2024 at the Medical University of Vienna (i.e., subsequent to recruitment of the derivation cohort). The same inclusion criteria as for the derivation cohort were applied; however, only patients with cACLD were included.

### 2.2 HVPG Measurement

Liver vein catheterization was performed to measure HVPG according to a standard operating procedure published previously [21], in line with national [18] and international [20] recommendations. In summary, under local anaesthesia and with the aid of ultrasound, a catheter introducer sheath was inserted into the right internal jugular vein. Subsequently, a specifically designed balloon catheter [22] was advanced into a large hepatic vein via the inferior vena cava under fluoroscopic guidance. Proper placement of the balloon catheter was confirmed by injecting contrast agent while the inflated balloon blocked the outflow of the cannulated hepatic vein. At least three measurements of both free and wedged hepatic vein pressure were taken to determine the HVPG.

### 2.3 Liver‐Related Events During the Follow‐Up Period

Liver‐related events denoting disease progression (vs. baseline) were recorded during the follow‐up period, i.e., the incidence of first decompensation, further decompensation, and liver‐related death (LRD). Patients were censored at the date of last clinical contact or liver transplantation in case no event occurred. In patients with compensated ACLD (cACLD), first decompensation was defined as the incidence of clinically evident ascites, variceal bleeding, or overt hepatic encephalopathy (HE). In patients with decompensated ACLD (dACLD), further decompensation was defined as the incidence of a second decompensation event or recurrent/refractory ascites, overt hepatic encephalopathy (HE), variceal bleeding, and/or the incidence of spontaneous bacterial peritonitis (SBP), hepatorenal syndrome acute kidney injury (HRS‐AKI), in line with Baveno VII consensus criteria [20]. Furthermore, the incidence of acute‐on‐chronic liver failure according to guidelines by the European Association of the Liver (EASL) was recorded [23].

### 2.4 Biomarker Measurement

Biomarkers of ECM remodelling were assessed in central venous blood samples obtained at HVPG measurement via the catheter introducer sheath. Laboratory parameters reflecting liver function and inflammatory parameters (C‐reactive protein, CRP; interleukin‐6, IL‐6; lipopolysaccharide binding protein, LBP), as well as ELF score were measured by the ISO‐certified Department of Laboratory Medicine, Medical University of Vienna, following the standardised protocols, as previously published [15, 24]. The Protein Fingerprint biomarkers nordicPRO‐C3, nordicPRO‐C4, nordicPRO‐C6 and nordicPRO‐C18L (reflecting formation of collagen III, IV, VI and XVIII), as well as nordicC3M, nordicC4M, and nordicC6Ma3 (reflecting degradation of collagen III, IV and VI) were assessed by competitive enzyme‐linked immunosorbent assay (ELISA). In the validation cohort, ELF, nordicPRO‐C3 and nordicPRO‐C6 were measured. Further details are provided in the Appendix S1.

### 2.5 Statistical Analysis

Statistical analyses were performed using R 4.3.2 (R Core Team, R Foundation for Statistical Computing, Vienna, Austria). Categorical variables are reported as absolute (n) and relative (%) frequencies, while continuous variables are displayed as mean ± standard error of the mean or median with interquartile range (IQR), as appropriate. Comparisons of continuous variables between two groups were performed using Mann–Whitney *U* or Student's *t*‐test based on their distribution, while three or more groups were compared by Wilcoxon Rank Sum test with Holm's multiple comparisons adjustment. Categorical variables were compared using chi squared or Fisher's exact test. Spearman's correlation coefficients (95% confidence interval) were calculated to assess the correlation between continuous variables. Kaplan–Meier curves and log‐rank test were used to visualise and compare the incidence of liver‐related events between groups: first decompensation for patients with compensated ACLD; further decompensation (as defined by Baveno VII [20]), ACLF or liver‐related death (composite endpoint) for patients with decompensated ACLD. Uni‐ and multivariable Cox regression models were performed to evaluate the independent prognostic value of parameters associated with liver‐related events [25]. Further information is delineated in the Appendix S1. The level of significance was set at a two‐sided *p*‐value < 0.05 for all analyses.

### 2.6 Ethical Considerations

The study was conducted in accordance with the principles of the Declaration of Helsinki and its amendments and approved by the local ethics committee of the Medical University of Vienna (EK 1262/2017). Patients provided written informed consent for participation in the prospective VICIS study (NCT03267615) and hepatic vein catheterization.

## 3 Results

### 3.1 Patient Characteristics

The study cohort comprised 232 patients with ACLD characterised by a median age of 59 [50–66] years, 64% male sex, and a median HVPG of 18 (13–21) mmHg and MELD of 11 (9–14) points. Patient characteristics are summarised in Table 1. Alcohol‐related liver disease (ALD; including those with metabolic dysfunction and alcohol‐related liver disease, MetALD) was the most common aetiology of liver disease, accounting for 45%, followed by viral hepatitis (18%) and metabolic dysfunction‐associated steatotic liver disease (10%). Regarding disease stages at baseline, 101 (44%) had compensated ACLD (cACLD) at the time point of HVPG measurement. Patients with decompensated ACLD (dACLD; *n* = 131, 56%) were distributed as follows: 78 had one previous decompensation event (i.e., first decompensation), and 53 had further decompensation according to Baveno VII criteria [20]. Notably, ALD represented the leading aetiology among patients with dACLD, while viral hepatitis was the most common aetiology in patients with cACLD.

**TABLE 1:** Patient characteristics in the overall cohort and patients stratified by compensated and decompensated ACLD.

	Overall cohort *N* = 232	cACLD *N* = 101	dACLD *N* = 131	*p*
Sex (*n*, %)				
F	82 (35.3%)	36 (35.6%)	46 (35.1%)	1.000
M	150 (64.7%)	65 (64.4%)	85 (64.9%)	
Age (years)	58.8 [50.1; 66.4]	59.2 [51.7; 68.2]	57.4 [50.0; 65.2]	0.422
HVPG (mmHg)	18.0 [13.0; 21.0]	13.0 [9.00; 18.0]	19.0 [16.0; 22.5]	< 0.001
Disease stage (*n*, %)				
cACLD	101 (43.5%)	101 (100%)	0 (0.00%)	< 0.001
First decompensation	78 (33.6%)	0 (0.00%)	78 (59.5%)	
Further decompensation	53 (22.8%)	0 (0.00%)	53 (40.5%)	
Aetiology (*n*, %)				
ALD	104 (44.8%)	23 (22.8%)	81 (61.8%)	< 0.001
Viral	42 (18.1%)	29 (28.7%)	13 (9.92%)	
ALD & Viral	15 (6.47%)	6 (5.94%)	9 (6.87%)	
MASLD	24 (10.3%)	21 (20.8%)	3 (2.29%)	
Cholestatic	8 (3.45%)	4 (3.96%)	4 (3.05%)	
Other	39 (16.8%)	18 (17.8%)	21 (16.0%)	
LSM (kPa)^a^	31.4 [19.3; 54.5]	23.4 [16.8; 35.3]	41.8 [24.5; 71.8]	< 0.001
Varices (*n*, %)				
None	85 (36.6%)	52 (51.5%)	33 (25.2%)	< 0.001
Small	64 (27.6%)	32 (31.7%)	32 (24.4%)	
Large	83 (35.8%)	17 (16.8%)	66 (50.4%)	
CTP stage (*n*, %)				
A	136 (58.6%)	92 (91.1%)	44 (33.6%)	< 0.001
B	76 (32.8%)	9 (8.91%)	67 (51.1%)	
C	20 (8.62%)	0 (0.00%)	20 (15.3%)	
MELD (points)	11.0 [9.00; 14.0]	9.00 [8.00; 12.0]	12.0 [10.0; 16.0]	< 0.001
WBC (G/L)	4.62 [3.31; 6.01]	4.79 [3.54; 6.22]	4.52 [3.23; 5.88]	0.094
Bilirubin (mg/dL)	1.08 [0.77; 1.71]	0.94 [0.65; 1.45]	1.22 [0.88; 1.97]	< 0.001
Albumin (mg/dL)	37.3 [33.6; 40.5]	39.9 [37.1; 42.2]	35.2 [31.3; 38.4]	< 0.001
CRP (mg/dL)	0.25 [0.12; 0.55]	0.20 [0.09; 0.39]	0.33 [0.16; 0.67]	< 0.001
IL‐6 (pg/mL)	7.19 [4.59; 12.6]	5.52 [3.36; 8.46]	8.83 [5.64; 15.0]	< 0.001
ELF score	11.3 (1.32)	10.7 (1.30)	11.8 (1.14)	< 0.001
TIMP1 (ng/mL)	313 [246; 435]	276 [230; 367]	365 [267; 485]	< 0.001
P3NP (ng/mL)	16.8 [11.6; 27.5]	12.8 [10.2; 19.8]	21.1 [13.5; 30.7]	< 0.001
HA (ng/mL)	207 [105; 403]	132 [69.6; 226]	285 [147; 506]	< 0.001
PRO‐C3 (ng/mL)	17.0 [11.8; 25.7]	14.6 [10.0; 21.0]	18.7 [13.5; 31.0]	0.001
PRO‐C4 (ng/mL)	223 [160; 303]	189 [146; 257]	237 [183; 330]	0.002
PRO‐C6 (ng/mL)	14.2 [10.7; 20.0]	11.7 [9.37; 15.4]	17.7 [12.5; 22.3]	< 0.001
PRO‐C18L (ng/mL)	2.95 [1.83; 4.55]	2.62 [1.65; 4.11]	3.13 [2.01; 4.88]	0.025
C3M (ng/mL)	14.0 [11.5; 18.0]	13.2 [10.8; 16.0]	15.2 [12.0; 19.8]	0.001
C4M (ng/mL)	30.5 [23.5; 38.8]	28.2 [21.6; 36.6]	33.0 [25.3; 40.0]	0.010
C6Ma3 (ng/mL)	0.32 [0.27; 0.42]	0.31 [0.26; 0.40]	0.34 [0.28; 0.43]	0.052
C3ratio	1.20 [0.81; 1.81]	1.12 [0.79; 1.62]	1.25 [0.82; 1.99]	0.218
C4ratio	7.37 [6.58; 8.29]	7.03 [6.32; 7.83]	7.64 [6.66; 8.58]	0.011
C6ratio	43.6 [32.3; 59.6]	36.1 [30.3; 52.4]	50.4 [36.9; 65.3]	< 0.001

*Note:* Statistical Analysis: Mann–Whitney *U* or Student's *t*‐test was used to compare continuous variables. Group comparisons of categorical variables were performed using Chi squared or Fisher's exact test.

Abbreviations: ALD, alcohol‐related liver disease; C3M, collagen type III metabolite; C3ratio, collagen type III ratio; C4M, collagen type IV metabolite; C4ratio, collagen type IV ratio; C6Ma3, collagen type VI metabolite; C6ratio, collagen type VI ratio; CRP, C‐reactive protein; CSPH, clinically significant portal hypertension; CTP, Child‐Turcotte‐Pugh; EASL, European Association for the Study of the Liver; ELF, enhanced liver fibrosis; HA, hyaluronic acid; HVPG, hepatic venous pressure gradient; IL‐6, interleukin‐6; LSM, liver stiffness measurement; MASLD, metabolic associated steatotic liver disease; MELD, model for end‐stage liver disease; P3NP, collagen type III N‐terminal propeptide; PH, portal hypertension; PRO‐C18L, collagen type XVIII propeptide; PRO‐C3, collagen type III propeptide; PRO‐C4, collagen type IV propeptide; PRO‐C6, collagen type VI propeptide; TIMP1, tissue inhibitor of metalloproteinases 1; WBC, white blood cell count.

^a^ LSM available in *n* = 202 (87%) of the cohort; cACLD: *n* = 92 (91%); dACLD: *n* = 110 (84%).

### 3.2 ECM Remodelling Biomarkers and Disease Severity

ECM biomarkers reflecting collagen formation and degradation of the interstitial matrix (collagen III: PRO‐C3, C3M; VI: PRO‐C6, C6Ma3) and basement membrane (collagen IV: PRO‐C4, C4M; XVIII: PRO‐C18L) were compared according to clinical disease stages based on previous decompensation events (Table 1). All biomarkers of collagen formation and degradation significantly increased in patients with dACLD (vs. cACLD; *p* < 0.05; except C6Ma3, *p* = 0.052).

**FIGURE 1:**
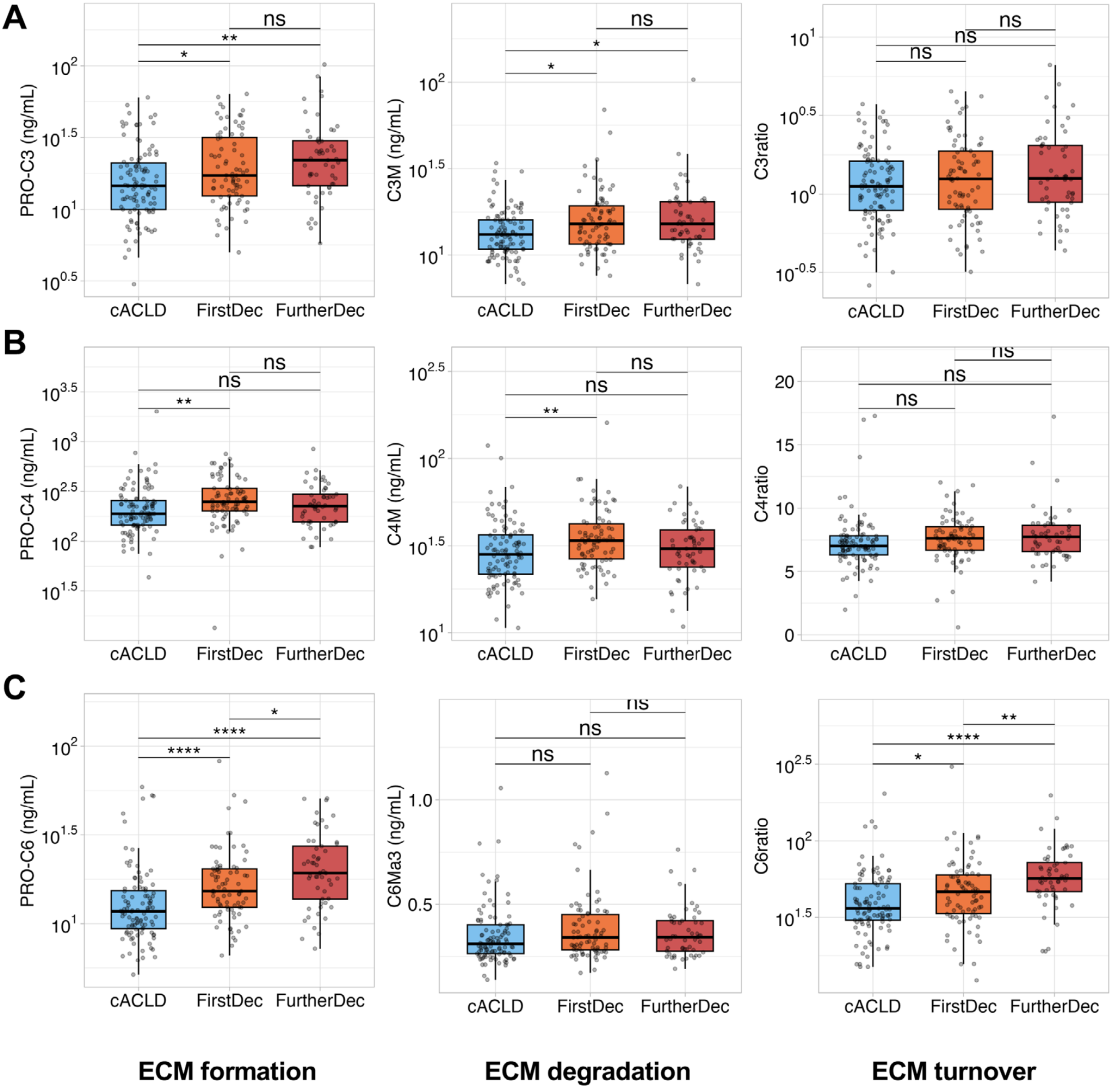
Biomarkers of extracellular matrix remodelling in patients stratified by different clinical disease stages of compensated and decompensated ACLD. ns, not significant; (*) *p* < 0.05; (**) *p* < 0.01; (****) *p* < 0.0001. Statistical analysis: Group comparisons were performed by Wilcoxon Rank Sum test with Holm's multiple comparisons adjustment. C3M, collagen type III metabolite; C3ratio, collagen type III ratio; C4M, collagen type IV metabolite; C4ratio, collagen type IV ratio; C6Ma3, collagen type VI metabolite; C6ratio, collagen type VI ratio; PRO‐C3, collagen type III propeptide; PRO‐C4, collagen type IV propeptide; PRO‐C6, collagen type VI propeptide.

When stratifying patients with dACLD by clinical sub‐stages (Figure 1), PRO‐C3 and PRO‐C6 showed a distinct increase in patients with dACLD. The collagen degradation biomarker C3M also increased in patients with dACLD, while no significant differences were observed for C6Ma3. The basement membrane remodelling biomarkers PRO‐C4 and C4M displayed a distinct increase in patients with first decompensation and a nonsignificant increase in further decompensation (adjusted for multiple testing). Based on the assumption that the relationship between corresponding collagen formation and degradation markers may reflect the equilibrium of ECM remodelling, ratios were calculated. C6ratio (PRO‐C6/C6Ma3) increased in patients with dACLD as compared to cACLD, while no meaningful difference in C3ratio and C4ratio was observed (Table 1, Figure 1).

**FIGURE 2:**
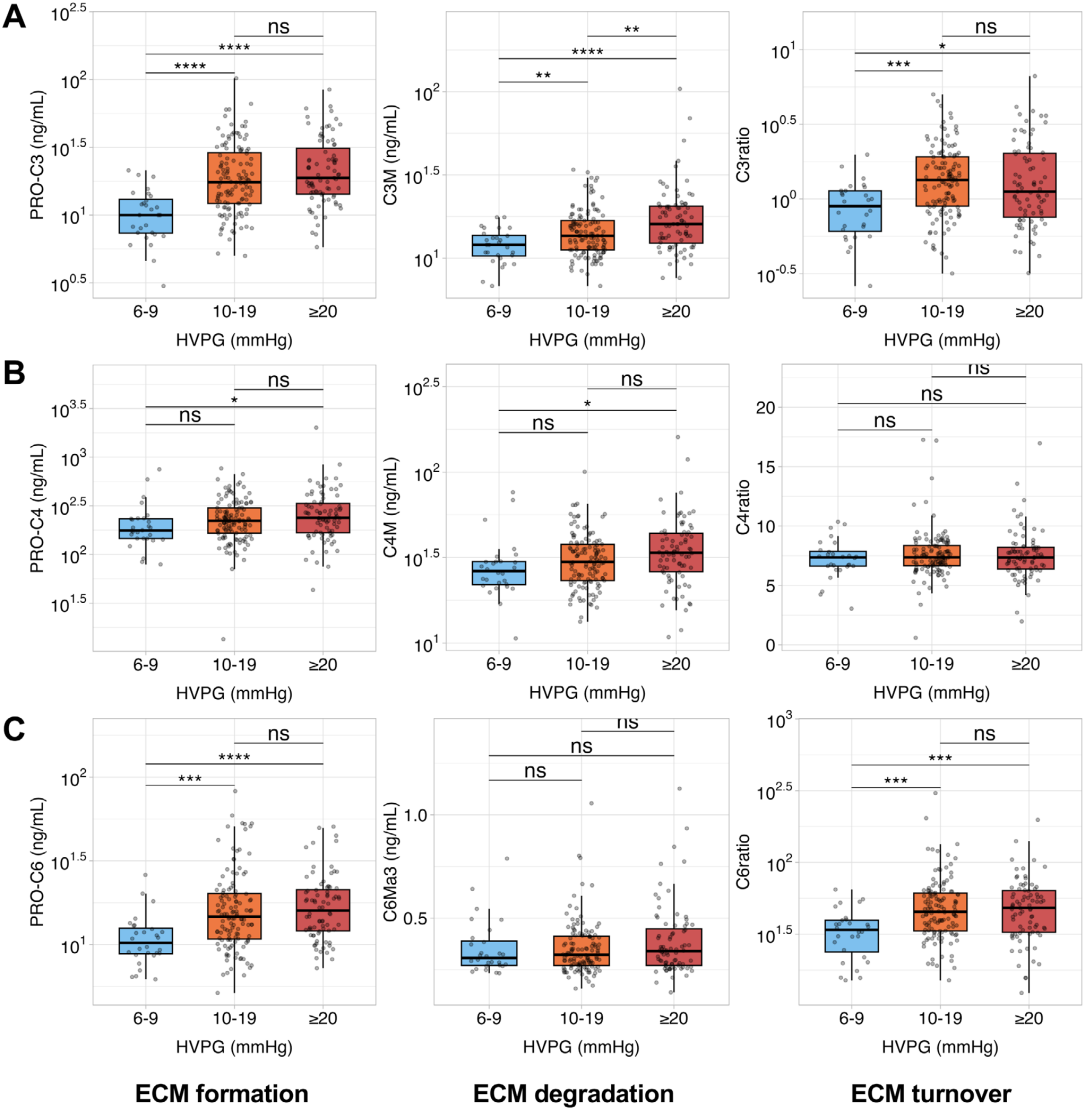
ECM remodelling biomarkers across patients stratified by HVPG. HVPG strata: *N* = 23 (12%) with 6–9 mmHg, *n* = 124 (53%) with 10–19 mmHg, and *n* = 80 (35%) with ≥ 20 mmHg. ns, not significant; (*) *p* < 0.05; (**) *p* < 0.01; (***) *p* < 0.001; (****) *p* < 0.0001. Statistical analysis: Group comparisons were performed by Wilcoxon Rank Sum test with Holm's multiple comparisons adjustment. C3M, collagen type III metabolite; C3ratio, collagen type III ratio; C4M, collagen type IV metabolite; C4ratio, collagen type IV ratio; C6Ma3, collagen type VI metabolite; C6ratio, collagen type VI ratio; HVPG, hepatic venous pressure gradient; PRO‐C3, collagen type III propeptide; PRO‐C4, collagen type IV propeptide; PRO‐C6, collagen type VI propeptide.

Subsequently, we investigated the relationship between fibrosis and PH as reflected by ECM biomarkers and HVPG, respectively (Figure 2, Figure S2). Patients were stratified into groups defined by HVPG: *n* = 23 (12%) with 6–9 mmHg, *n* = 124 (53%) with 10–19 mmHg, and *n* = 80 (35%) with ≥ 20 mmHg. As mentioned above, a subset of ECM biomarkers was already reported previously in a broadly overlapping cohort within a different research context [14]. Regarding interstitial matrix biomarkers, PRO‐C3 and C3M, and interestingly, also the C3 ratio increased across HVPG strata. PRO‐C6 displayed a significant increase in patients with an HVPG ≥ 10 mmHg, while no dynamics of C6Ma3 were observed. Regarding basement membrane biomarkers, significant increases of PRO‐C4 and C4M were restricted to patients with an HVPG ≥ 20 mmHg. PRO‐C18L showed no clinically meaningful pattern throughout disease stages or HVPG strata (Figure S2). Importantly, ELF score gradually increased in patients with first and further decompensation, as compared to patients with cACLD, and across HVPG groups (Figure S2).

Finally, liver stiffness measurement (LSM; assessed by FibroScan) was available in 202 (87%) patients at the timepoint of HVPG measurement. Comparison of ECM biomarkers by LSM‐based stratification of patients showed analogous dynamics with increasing LSM values to HVPG groups (Figures S2 and S3).

### 3.3 ECM Remodelling Biomarkers and Established Parameters of Disease Severity and Prognosis

Correlation analyses between ECM biomarkers and parameters indicating disease severity and prognosis were performed. In the overall cohort, moderate (ELF: *r* = 0.507, *p* < 0.001) or weak positive correlations between HVPG and biomarkers of collagen formation (PRO‐C3: Spearman's *r* = 0.295; PRO‐C4: *r* = 0.172; PRO‐C6: *r* = 0.252; all *p* < 0.01; PRO‐C18L: *r* = 0.124, *p* = 0.080), and either weak or no correlations between HVPG and collagen degradation biomarkers or ECM biomarker ratios for collagen III, IV and VI were observed (Figure S4).

Subsequently, correlation matrices assessing the relationships between ECM biomarkers, PH (HVPG), and indicators of liver function (MELD and albumin) as well as systemic inflammation (CRP and IL‐6) were created (Table S1, Figure S5). PRO‐C3/‐C4/‐C6 levels correlated directly with their respective degradation products (C3M: *r* = 0.369; C4M: *r* = 0.894; C6Ma3: *r* = 0.360; all *p* < 0.001). PRO‐C3 and P3NP (procollagen‐3 N‐terminal peptide, i.e., a collagen III formation biomarker incorporated in ELF score) displayed a strong direct correlation (*r* = 0.880, *p* < 0.001).

In general, ECM biomarkers displayed a statistically significant link to albumin levels (negative correlation; particularly ELF *r* = −0.671 and PRO‐C6 *r* = −0.469, *p* < 0.001) and MELD score (particularly ELF *r* = 0.534, PRO‐C6 *r* = 0.364; *p* < 0.001), CRP and IL‐6 levels. C3/C4/C6 ratios did not display an incrementing correlation strength as compared to single biomarkers. Linear regression models assessing potential influencing factors on collagen degradation are delineated in the Appendix S1.

**FIGURE 3:**
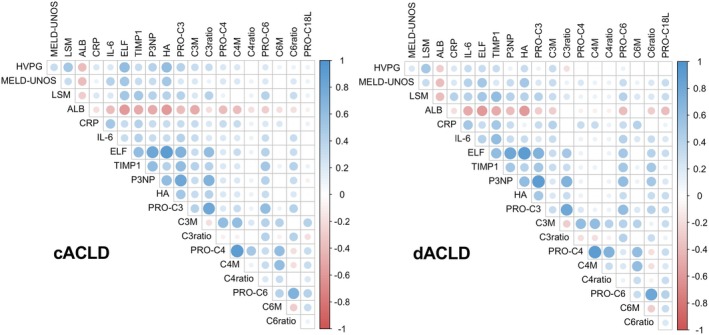
Correlation matrices: The relationship between ECM formation and degradation biomarkers, disease severity and inflammation in compensated and decompensated ACLD. Statistical analysis: Spearman's correlation coefficient was calculated to assess the association between continuous variables. c/dACLD, compensated/decompensated advanced chronic liver disease; C3M, collagen type III metabolite; C3ratio, collagen type III ratio; C4M, collagen type IV metabolite; C4ratio, collagen type IV ratio; C6Ma3, collagen type VI metabolite; C6ratio, collagen type VI ratio; CRP, C‐reactive protein; ELF, enhanced liver fibrosis score; HA, hyaluronic acid; HVPG, hepatic venous pressure gradient; IL‐6, interleukin‐6; LSM, liver stiffness measurement; MELD, model for end‐stage liver disease; P3NP, collagen type III N‐terminal propeptide; PRO‐C18L, collagen type XVIII formation marker; PRO‐C3, collagen type III propeptide; PRO‐C4, collagen type IV propeptide; PRO‐C6, collagen type VI propeptide; TIMP1, tissue inhibitor of metalloproteinases 1.

Based on previous observations on distinct correlation patterns of fibrosis biomarkers within cACLD and dACLD [15], and the observation that fibrogenesis biomarkers were elevated in patients stratified by disease stage or HVPG strata, but exhibited only weak direct correlations within the overall cohort, correlation matrices within cACLD and dACLD subgroups were created (Figure 3, Tables S3 and S4). In patients with cACLD, the correlation strength between HVPG and ELF (*r* = 0.609, *p* < 0.001), PRO‐C3 (*r* = 0.443, *p* < 0.001), as well as PRO‐C4 (*r* = 0.199, *p* < 0.05) increased, while no significant association between HVPG and PRO‐C6 or PRO‐C18L was observed. In patients with dACLD, correlation strength between HVPG and fibrogenesis biomarkers either markedly decreased (ELF *r* = 0.278, *p* < 0.001), or the correlation did not attain statistical significance (PRO‐C3/‐C4/‐C6/‐C18L). Notably, the opposite pattern was observed for MELD, which showed no meaningful link to ECM biomarkers in cACLD (except ELF score, *r* = 0.476, *p* < 0.001), while displaying a significant link to all available fibrogenesis biomarkers in dACLD (Table S4). Finally, ratios of collagen formation and degradation biomarkers displayed no incremental information regarding the correlation with other measures of disease severity and inflammation, as compared to individual biomarkers.

Finally, most ECM formation biomarkers as well as HVPG showed a moderate significant correlation with LSM: *r* = 0.504 for PRO‐C3, *r* = 0.445 for PRO‐C6, *r* = 0.570 for ELF, *r* = 0.599 for HVPG (all *p* < 0.05; Table S1). Conversely, weak or no direct relationships with LSM were observed for PRO‐C4, PRO‐C18L and ECM degradation biomarkers. Correlation patterns were similar when performing correlation analyses in subgroups stratified into cACLD and dACLD (Tables S3 and S4).

### 3.4 ECM Remodelling Biomarkers and Disease Progression

The prognostic value of ECM remodelling biomarkers towards disease progression was analysed, considering that both fibrogenesis and fibrolysis may indicate disease activity in ACLD. During the transplant‐free follow‐up period of 29.0 (12.1–43.7) months, 93 (40%) patients experienced an event of disease progression as defined by the composite endpoint of first or further decompensation, ACLF, or liver‐related death.

**FIGURE 4:**
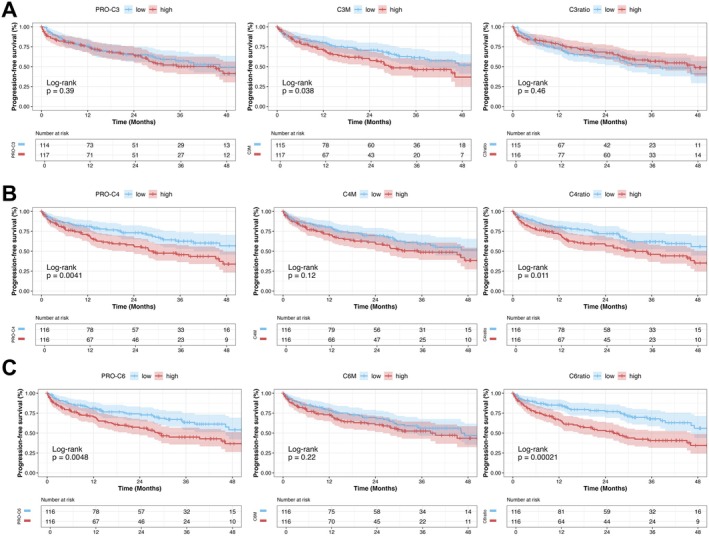
Incidence of liver‐related events during the follow‐up period. Statistical analysis: Kaplan–Meier curves indicating disease progression‐free follow‐up (composite endpoint: First/further decompensation, ACLF, and liver related death) were compared by log‐rank test. Groups are defined as either below “low” or above “high” median biomarker levels. C3M, collagen type III metabolite; C3ratio, collagen type III ratio; C4M, collagen type IV metabolite; C4ratio, collagen type IV ratio; C6Ma3, collagen type VI metabolite; C6ratio, collagen type VI ratio; PRO‐C3, collagen type III propeptide; PRO‐C4, collagen type IV propeptide; PRO‐C6, collagen type VI propeptide.

First, Kaplan–Meier curves either stratifying patients into groups by median or tercile biomarker levels were plotted (Figure 4; Figures S9–S15). In the full cohort, stratification by median and terciles indicated that patients with high PRO‐C4, PRO‐C6, PRO‐C18L and ELF levels are at higher risk of disease progression (log‐rank; all *p* < 0.01). Notably, patients within the highest tercile of PRO‐C4 (≥ 233 ng/mL) had an extraordinarily high risk of adverse liver‐related events (Figure S9).

Subsequently, the incidence of first hepatic decompensation in patients with cACLD (22 events, 22%, follow‐up 31.4 [13.6–43.3] months) was compared in patients stratified by median or terciles (Figures S10, S11, S14 and S15). Of note, one patient with liver‐related death associated with metastasized hepatocellular carcinoma (diagnosed during follow‐up) without hepatic decompensation was censored for this analysis. Importantly, cACLD patients with low ELF, PRO‐C4 and PRO‐C6 levels had a lower risk of first hepatic decompensation (all log‐rank *p* < 0.05), while PRO‐C3 and PRO‐C18L (all *p* > 0.10) did not show prognostic value. Notably, collagen degradation markers or their ratios did not provide meaningful information on the risk of first decompensation.

In patients with dACLD, neither stratification by median or tercile ECM formation or degradation biomarker levels or ELF score provided a significant difference regarding the risk of further decompensation, ACLF, or liver‐related death (70 events, follow‐up 28.2 [11.4–44.8] months; Figures S12–S15).

Finally, Cox regression models including HVPG, MELD, albumin and disease stage (presence of dACLD) at baseline were performed to determine the independent prognostic value of ECM remodelling biomarkers for adverse liver‐related events (Table 2). In accordance with Kaplan–Meier curves, PRO‐C4 (per 100 ng/mL: HR 1.13, 1.07–1.19, < 0.001), PRO‐C6 (per ng/mL: HR 1.02, 1.00–1.03, *p* = 0.056), and ELF (per point: HR 1.41, 1.22–1.63, *p* < 0.001) were linked to disease progression on univariate analyses. Next to HVPG and albumin, multivariate models indicated an independent predictive value of PRO‐C4 (aHR per 100 ng/mL: 1.22, 1.09–1.36, *p* < 0.001; i.e., higher risk with higher values) as well as the fibrolysis markers C3M (aHR per ng/mL: 0.98, 0.96–0.99, *p* = 0.044; i.e., reduced risk with higher values) and C4M (aHR per ng/mL: 0.98, 0.96–1.00, *p* = 0.084; i.e., reduced risk with higher values) for disease progression.

**TABLE 2:** Cox regression models for the prediction of disease progression (overall cohort).

Overall cohort	Univariable		Multivariable (first model)		Multivariable (last model)	
	HR (95% CI)	*p*	aHR (95% CI)	*p*	aHR (95% CI)	*p*
C3‐biomarkers						
Age (per year)	1.01 (0.99–1.03)	0.353	1.01 (0.99–1.03)	0.308	—	—
HVPG (mmHg)	1.08 (1.05–1.12)	**< 0.001**	1.06 (1.02–1.10)	**0.001**	1.06 (1.02–1.09)	**0.001**
MELD (points)	1.07 (1.03–1.12)	**0.001**	0.99 (0.93–1.05)	0.759	—	—
Albumin (g/L)	0.90 (0.86–0.93)	**< 0.001**	0.91 (0.87–0.96)	**< 0.001**	0.92 (0.88–0.97)	**< 0.001**
dACLD (yes)	2.83 (1.79–4.50)	**< 0.001**	1.63 (0.95–2.80)	0.073	1.58 (0.93–2.70)	0.092
PRO‐C3 (ng/mL)	1.00 (0.99–1.01)	0.561	0.99 (0.98–1.01)	0.325	—	—
C3M (ng/mL)	1.00 (0.98–1.02)	0.704	0.98 (0.96–1.00)	0.074	0.98 (0.96–0.99)	**0.044**
C3ratio	0.97 (0.79–1.19)	0.762	—	—	—	—
C4‐biomarkers						
Age (per year)	1.01 (0.99–1.03)	0.353	1.01 (0.99–1.03)	0.328	—	—
HVPG (mmHg)	1.08 (1.05–1.12)	**< 0.001**	1.05 (1.01–1.09)	**0.022**	1.05 (1.01–1.09)	**0.018**
MELD (points)	1.07 (1.03–1.12)	**0.001**	1.00 (0.95–1.06)	0.916	—	—
Albumin (g/L)	0.90 (0.86–0.93)	**< 0.001**	0.93 (0.88–0.99)	**0.012**	0.93 (0.89–0.98)	**0.005**
dACLD (yes)	2.83 (1.79–4.50)	**< 0.001**	1.61 (0.95–2.75)	0.077	1.62 (0.96–2.74)	0.073
PRO‐C4 (per 100 ng/mL)	1.13 (1.07–1.19)	**< 0.001**	1.20 (1.08–1.34)	**< 0.001**	1.22 (1.09–1.36)	**< 0.001**
C4M (ng/mL)	1.01 (0.99–1.02)	0.229	0.99 (0.97–1.00)	0.097	0.98 (0.96–1.00)	0.084
C4ratio	1.14 (1.05–1.23)	**0.002**	—	—	—	—
C6‐biomarkers						
Age (per year)	1.01 (0.99–1.03)	0.353	1.01 (0.99–1.03)	0.258	—	—
HVPG (mmHg)	1.08 (1.05–1.12)	**< 0.001**	1.05 (1.01–1.09)	**0.022**	1.05 (1.01–1.09)	**0.018**
MELD (points)	1.07 (1.03–1.12)	**0.001**	1.00 (0.94–1.06)	0.936	—	—
Albumin (g/L)	0.90 (0.86–0.93)	**< 0.001**	0.93 (0.88–0.98)	**0.010**	0.93 (0.89–0.98)	**0.004**
dACLD (yes)	2.83 (1.79–4.50)	**< 0.001**	1.56 (0.90–2.70)	0.115	1.61 (0.94–2.74)	0.082
PRO‐C6 (ng/mL)	1.02 (1.00–1.03)	0.056	1.01 (0.98–1.03)	0.603	—	—
C6Ma3 (ng/mL)	2.17 (0.44–10.6)	0.340	1.04 (0.15–7.33)	0.968	—	—
C6ratio	1.00 (0.99–1.01)	0.196	—	—	—	—
ELF score						
Age (per year)	1.01 (0.99–1.03)	0.353	1.01 (0.99–1.03)	0.286	—	—
HVPG (mmHg)	1.08 (1.05–1.12)	**< 0.001**	1.04 (1.00–1.09)	**0.027**	1.05 (1.01–1.09)	**0.018**
MELD (points)	1.07 (1.03–1.12)	**0.001**	1.00 (0.94–1.06)	0.929	—	—
Albumin (g/L)	0.90 (0.86–0.93)	**< 0.001**	0.93 (0.88–0.99)	**0.027**	0.93 (0.89–0.98)	**0.004**
dACLD (yes)	2.83 (1.79–4.50)	**< 0.001**	1.60 (0.94–2.72)	0.081	1.61 (0.94–2.74)	0.082
ELF score	1.41 (1.22–1.63)	**< 0.001**	1.06 (0.85–1.32)	0.613	—	—
PRO‐C18L						
Age (per year)	1.01 (0.99–1.03)	0.353	1.01 (0.98–1.03)	0.265	—	—
HVPG (mmHg)	1.08 (1.05–1.12)	**< 0.001**	1.04 (1.00–1.09)	**0.022**	1.05 (1.01–1.09)	**0.018**
MELD (points)	1.07 (1.03–1.12)	**0.001**	1.00 (0.94–1.06)	0.903	—	—
Albumin (g/L)	0.90 (0.86–0.93)	**< 0.001**	0.93 (0.88–0.98)	**0.010**	0.93 (0.89–0.98)	**0.004**
dACLD (yes)	2.83 (1.79–4.50)	**< 0.001**	1.60 (0.94–2.74)	0.083	1.61 (0.94–2.74)	0.082
PRO‐C18L (ng/mL)	1.01 (0.97–1.04)	0.719	0.99 (0.95–1.03)	0.636	—	—

*Note:* Statistical Analysis: Uni‐ and multivariable Cox regression analyses of factors associated with first/further hepatic decompensation, development of ACLF and liver‐related death (composite endpoint; *n* = 93 events). *p*‐values below 0.05 are highlighted in bold.

Abbreviations: aHR, adjusted hazard ratio; C3M, collagen type III metabolite; C3ratio, collagen type III ratio; C4M, collagen type IV metabolite; C4ratio, collagen type IV ratio; C6Ma3, collagen type VI metabolite; C6ratio, collagen type VI ratio; ELF, enhanced liver fibrosis score; HVPG, hepatic venous pressure gradient; MELD, model for end‐stage liver disease; PRO‐C18L, collagen type XVIII propeptide; PRO‐C3, collagen type III propeptide; PRO‐C4, collagen type IV propeptide; PRO‐C6, collagen type VI propeptide.

Cox regression models performed in cACLD and dACLD subgroups individually are shown in Tables S5 and S6. In summary, ECM biomarkers were associated with first decompensation in cACLD; however, only HVPG and albumin remained independently associated with this endpoint in multivariate analyses. In contrast, ECM biomarkers showed no association with disease progression (further decompensation, ACLF, LRD) in patients with stable dACLD.

Exploratory evaluation of time‐dependent area‐under‐the‐receiver operating characteristics (AUROC) confirmed that the discriminative ability of ECM formation biomarkers for first decompensation in cACLD was largely similar to that of HVPG, particularly PRO‐C4 and ELF score (Figures S16 and S17). However, we abstained from more extensive head‐to‐head comparisons due to limited sample size/number of events in the cACLD subgroup. Contrarily, ECM formation biomarkers could not discriminate between dACLD patients with or without disease progression.

### 3.5 Validation in Independent Cohort With Compensated ACLD

Based on the observation that selected ECM formation biomarkers were predictive for disease progression in cACLD (but not dACLD), ELF, PRO‐C3 and PRO‐C6 were measured in an independent validation cohort that underwent HVPG measurement to investigate whether these biomarkers showed similar predictive value for first decompensation. Briefly, 185 patients with cACLD were included in the validation cohort and showed similar clinical characteristics as compared to the validation cohort (Table S7).

During a median follow‐up of 24.2 (15.1–37.7) months, 36 (19.5%) of the validation cohort developed first hepatic decompensation, representing a similar follow‐up time and decompensation rate compared to the derivation cohort. Stratification by median biomarker levels showed a significantly higher risk for first hepatic decompensation in patients with high fibrogenesis biomarker levels. Similar results were observed when stratifying patients by median LSM (available in 98% of the cohort; Figure S18).

Furthermore, Cox regression models for the validation cohort showed comparable univariate hazard ratios for ELF (HR per point: 1.59, 1.28–1.96, *p* < 0.001), PRO‐C3 (HR per ng/mL: 1.04, 1.01–1.06, *p* = 0.004), and PRO‐C6 (HR per point: 1.02, 1.00–1.05, *p* = 0.094; Table S8), as well as time‐dependent AUROC performance (Figure S19) for prediction of first decompensation, as compared to the derivation cohort. Notably, ECM formation biomarkers had similar predictive value for disease progression as LSM in the validation cohort.

## 4 Discussion

The present study investigated the relationship between biomarkers reflecting the dynamic process of ECM formation and degradation and disease severity and prognosis in ACLD. To this end, we measured an extensive panel of blood biomarkers of ECM remodelling in 232 prospectively recruited patients undergoing HVPG measurement with extensive clinical characterisation, and recorded adverse liver‐related events during follow‐up. Importantly, the biomarker panel not only included ELF score and its individual components capturing different aspects of fibrogenesis [26], but also biomarkers reflecting the formation of collagen III, IV and VI and their corresponding collagen degradation products [6]. Furthermore, a propeptide of collagen XVIII—the latter functioning as a precursor of the angiogenesis inhibitor endostatin [27]—was measured.

The relevance of exploring ECM biomarkers in patients with ACLD is emphasised by several considerations. First of all, blood biomarkers of liver fibrosis were primarily developed to discriminate histological fibrosis severity by measuring molecules associated with collagen formation or other aspects of ECM remodelling in the liver [6]. Concordantly, ELF score [9, 10, 28, 29], as well as PRO‐C3, PRO‐C6 and C3M were linked to histological fibrosis stages within different etiologies of chronic liver disease [11, 12, 13, 30]. However, the mentioned studies predominantly included patients with pre‐cirrhotic liver disease, investigated only partially overlapping biomarker panels, and/or did not assess the relationship with clinical outcomes. In contrast, the present study cohort exclusively included patients with ACLD, i.e., within the spectrum of advanced liver fibrosis and cirrhosis. In patients with dACLD, bursts of systemic inflammation in the context of AD and/or ACLF may temporarily impact ECM remodelling, and thus, impede investigations between ECM remodelling and clinical outcomes [14, 31, 32]. Therefore, we exclusively studied ACLD patients in a clinically stable condition at the time of sampling—i.e., excluding patients with AD or ACLF, which were specifically investigated in other studies [31, 32]. Table S9 summarises relevant aspects regarding study design, biomarker selection and results in mentioned previous studies.

We have previously demonstrated that HSC activation assessed by liver immunohistochemistry (i.e., alpha smooth muscle actin‐positive area) is linked to ECM biomarker levels in patients with ACLD [14]. In ACLD, however, the potential clinical use of biomarkers reflecting essential pathophysiological processes such as ECM formation shifts from quantifying fibrosis to providing information on PH (i.e., HVPG) or predicting liver‐related events [33, 34, 35]. To this end, we observed that biomarkers associated with ECM formation (fibrogenesis; i.e., ELF, PRO‐C3, PRO‐C4, PRO‐C6 and PRO‐C18L) increased in patients with CSPH and dACLD, substantiating that the process of fibrogenesis still displays considerable stage‐dependent variability in ACLD.

Notably, we observed that the direct correlation between HVPG and fibrogenesis biomarkers displayed weak to moderate strength in the study cohort. This may be explained by the exclusive selection of patients with ACLD and the high prevalence of CSPH, which weakens the correlation of fibrosis biomarkers with PH [15, 36], as the relative contribution of fibrosis to PH decreases in advanced disease [37, 38]. This consideration is supported by correlation analyses restricted to patients with either cACLD or dACLD in our study showing stronger correlations between ECM biomarkers and HVPG in cACLD, and data from previous studies that also included individuals without ACLD (HVPG < 6 mmHg) showing a stronger correlation between PRO‐C3 and HVPG [39, 40]. Notably, the increase of ECM biomarkers in dACLD might be predominantly driven by pathomechanisms other than PH, such as systemic inflammation [14]. All things considered, our findings indicate that elastography‐based models (e.g., ANTICIPATE±NASH [41] and NICER [42]) or more capable blood‐based noninvasive tests (NIT; e.g., VITRO [33, 43]) are preferred over ECM biomarkers for estimating the probability of CSPH in cACLD [44].

Potential strong points of ECM biomarkers relate, on the one hand, to the possibility of performing repeated measurements that may reflect the clinical trajectory of liver disease, and on the other hand, to the availability of biomarkers reflecting ECM degradation as an indicator of fibrosis regression [45]. For example, a previous study demonstrated that an increase in PRO‐C3 mirrors histological fibrosis progression when performing a repeated biopsy [30], and conversely, the ELF score decreased after hepatitis C virus (HCV) treatment [46]. Importantly, the dynamics of liver stiffness add limited prognostic information, which may be explained by limited repeatability, translating into a high minimal detectable change [47]; the high precision on ELF [48] and PRO‐C3 [49] suggests that ECM remodelling biomarkers may be capable of overcoming this limitation. Moreover, ECM remodelling biomarkers may help distinguish “hot” fibrosis that is characterised by active inflammation and immune cell infiltration, from “cold” fibrosis that is characterised by inactive scar tissue, by reflecting differences in ECM composition and degradation activity [50].

In another context, previous studies suggested that the ratios between collagen formation and degradation biomarkers (e.g., C3 ratio) provide information on the dynamic equilibrium of ECM remodelling. For example, C3 ratio reflected either disease progression [31] or successful HCV eradication [51]. In our study, biomarkers were measured at a single timepoint, and thus, we cannot evaluate the relevance of biomarker trajectories. However, the calculation of C3‐/C4‐/C6‐ratios on a cross‐sectional level provided no additional value towards reflection of disease stage or PH severity as well as outcome prediction (discussed below) in our patient cohort.

Importantly, our study investigated the prognostic value of ECM biomarkers for disease progression in patients with ACLD. To this end, disease progression was defined as first/further decompensation or ACLF (in addition to liver‐related death), which seems reasonable considering their well‐documented detrimental impact on mortality risk [52, 53]. Previous work had documented that ECM biomarkers such as ELF score, PRO‐C3, or PRO‐C6 are associated with adverse liver‐related events in chronic liver disease. Importantly, previous studies either included a significant number of patients with mild fibrosis (i.e., easily classifiable patients at negligible risk), or were restricted to either cACLD or ACLF, respectively [8, 16, 32, 54].

We observed differences in prognostic value of individual biomarkers when stratifying patients into cACLD and dACLD subgroups, suggesting that the prognostic relevance of the underlying pathophysiological processes varies according to disease stage. Importantly, we found that ELF score, PRO‐C3, PRO‐C4 and PRO‐C6 were prognostic for the first decompensation in patients with cACLD. However, only HVPG and albumin remained independently associated with this endpoint in multivariate analysis. Nevertheless, exploratory time‐dependent AUROC analyses suggested that the prognostic performance of individual ECM biomarkers is (almost) comparable to that of HVPG in cACLD, the (minimally) invasive gold standard for PH assessment. The latter serves not only as a prognostic but also as a predictive biomarker, as CSPH identifies the population of cACLD patients who benefit from NSBB/carvedilol therapy for preventing hepatic decompensation [55]. We acknowledge that our study may be limited by the high CSPH prevalence in our cohort and the single‐center design. Nevertheless, the predictive value of ECM formation biomarkers in cACLD (ELF, PRO‐C3 and PRO‐C6) was validated in an independent cohort undergoing HVPG measurement that was recruited after the derivation cohort, thus, formally representing independent validation. Still, the present study warrants future (multicenter) studies with repeated biomarker assessment to determine the best ECM biomarkers for disease monitoring and prognostication in ACLD. Nevertheless, it extends the observation that noninvasive tests for PH are similarly predictive of clinical outcomes in cACLD, as compared to HVPG by providing data in ECM biomarkers [34]. Conversely, in patients with dACLD, the prognostic value of individual fibrogenesis biomarkers was either weak or nonsignificant, which is in line with the concept that disease progression in this patient group is dominated by other pathophysiological factors, such as systemic inflammation [56].

In conclusion, ECM remodelling biomarkers are linked to PH and ACLD severity; however, their predictive value for disease progression is mostly restricted to cACLD. Future studies should investigate the time‐dependent dynamics of ECM biomarkers (i.e., paired measurements) in patients with cACLD undergoing etiological therapy and experiencing either progression or recompensation of dACLD to understand how repeated measurements of ECM remodelling biomarkers reflect the clinical trajectory of ACLD. Furthermore, a head‐to‐head comparison between minimally invasive prognostic indicators such as HVPG and noninvasive models involving ECM biomarkers should be conducted in larger cACLD cohorts.

## Author Contributions

**Benedikt Simbrunner:** conceptualization, writing – original draft, investigation, visualization, methodology, data curation. **Ida Falk Villesen:** data curation, methodology, visualization, investigation, conceptualization, writing – review and editing. **Georg Semmler:** writing – review and editing, data curation. **Lorenz Balcar:** data curation, writing – review and editing, methodology. **Georg Kramer:** data curation, writing – review and editing. **Joana Almeida Calvão:** data curation, writing – review and editing. **Benedikt Silvester Hofer:** writing – review and editing, data curation. **Mathias Jachs:** writing – review and editing, data curation. **Lukas Hartl:** writing – review and editing, data curation. **Jurij Maurer:** writing – review and editing, data curation. **Bernhard Scheiner:** writing – review and editing, data curation. **Kerstin Zinober:** methodology, data curation. **Rodrig Marculescu:** data curation, writing – review and editing, methodology. **Michael Trauner:** writing – review and editing, data curation, resources. **Morten Karsdal:** writing – review and editing, data curation. **Thomas Reiberger:** methodology, writing – review and editing, data curation, project administration, resources. **Mattias Mandorfer:** project administration, supervision, data curation, methodology, conceptualization, writing – original draft, investigation, resources. **Diana Julie Leeming:** conceptualization, data curation, project administration, resources, writing – review and editing, methodology, supervision, investigation.

## Conflicts of Interest

BeSi has received travel support from AbbVie, Gilead and Falk. I.F.V. was a full‐time employee at Nordic Bioscience at the time of biomarker measurements. M.J. has served as a speaker and consultant for Gilead. BeSc served as a speaker for AstraZeneca and Eisai and received grant support from AstraZeneca and Eisai as well as travel support from AbbVie, AstraZeneca, Ipsen and Gilead. M.T. received grant support from Albireo, Alnylam, Cymabay, Falk, Genentech, Gilead, Intercept, MSD, Takeda and UltraGenyx; honoraria for consulting from AbbVie, Albireo, Agomab, Boehringer Ingelheim, BiomX, Chemomab, Dexoligo Therapeutics, Falk, Genfit, Gilead, GSK, Hightide, Intercept, Ipsen, Janssen, MSD, Novartis, Phenex, Pliant, Regulus, Siemens and Shire; speaker fees from Albireo, Boehringer Ingelheim, Bristol‐Myers Squibb, Falk, Gilead, Ipsen, Intercept, MSD and Madrigal; as well as travel support from AbbVie, Falk, Gilead, Janssen and Intercept. He is also coinventor of patents on the medical use of 24‐norursodeoxycholic acid filed by the Medical University of Graz. M.K. is a full‐time employee and stock owner at Nordic Bioscience and among the original inventors and patent holders of the Nordic Bioscience biomarkers. T.R. received grant support from AbbVie, Boehringer Ingelheim, Gilead, Intercept/Advanz Pharma, MSD, Myr Pharmaceuticals, Philips Healthcare, Pliant, Siemens and W. L. Gore & Associates; speaking honoraria from AbbVie, Echosens, Gilead, Intercept/Advanz Pharma, Roche, MSD and W. L. Gore & Associates; consulting/advisory board fees from AbbVie, Astra Zeneca, Bayer, Boehringer Ingelheim, Gilead, Intercept/Advanz Pharma, MSD, Resolution Therapeutics and Siemens; and travel support from AbbVie, Boehringer Ingelheim, Falk, Gilead and Roche. M.M. served as a speaker and/or consultant and/or advisory board member for AbbVie, AstraZeneca, Echosens, Eli Lilly, Falk, Gilead, Ipsen, Takeda and W. L. Gore & Associates and received grant support from Echosens as well as travel support from AbbVie and Gilead. D.J.L. is a full‐time employee and stock owner at Nordic Bioscience and among the original inventors and patent holders of the Nordic Bioscience biomarkers. All other authors declare no conflicts of interest.

## Supporting information


**Appendix S1:** apt70407‐sup‐0001‐AppendixS1.docx.

## Data Availability

The data that support the findings of this study are available from the corresponding author upon reasonable request.
