# Supplementary material for: Biomarkers of Extracellular Matrix Remodelling Are Linked to Severity and Outcome of Advanced Chronic Liver Disease
Source: Aliment Pharmacol Ther. 2025 Oct 12;63(5):648–61. doi: 10.1111/apt.70407 (PMC12904196; doi:10.1111/apt.70407)
Supplement: Supplementary file 1 — Appendix S1: apt70407‐sup‐0001‐AppendixS1.docx. [file APT-63-648-s001.docx]

**SUPPLEMENTARY MATERIAL**

**Title: Biomarkers of extracellular matrix remodeling are linked to severity and outcome of advanced chronic liver disease**

**Table of contents**

[Supplementary methods 2](#_Toc210063659)

[Statistical analysis 2](#_Toc210063660)

[Time-dependent area-under-the-receiver operating characteristics 2](#_Toc210063661)

[Biomarker measurements 2](#_Toc210063662)

[Supplementary results 3](#_Toc210063663)

[Linear regression 3](#_Toc210063664)

[Supplementary figures 4](#_Toc210063665)

[Supplementary tables 23](#_Toc210063666)

[References (used in the supplementary material) 38](#_Toc210063667)

# Supplementary methods

## Statistical analysis

Impact of biomarkers of interest on liver-related outcomes was assessed using Cox regression analyses using the 'survival' package in R. Clinical events of interest were chosen according to disease stage: first decompensation for patients with compensated ACLD; further decompensation (as defined by Baveno VII [1]), ACLF or liver-related death (composite endpoint) for patients with decompensated ACLD. We included all parameters into univariable Cox regression models. In a first step, all variables were included into the multivariable Cox regression model. Variable selection of the 'final' model was based on backward elimination eliminating variables with p > 0.157 [2].

## Time-dependent area-under-the-receiver operating characteristics

Time-dependent area-under-the-receiver operating characteristics (AUROC) curves were drawn using the ‘timeROC’ package in R. Clinical events of interest were chosen according to disease stage: first decompensation for patients with compensated ACLD; further decompensation (as defined by Baveno VII [1]), ACLF or liver-related death (composite endpoint) for patients with decompensated ACLD. In the overall cohort, a composite endpoint of all above-mentioned clinical events was chosen.

## Biomarker measurements

A competitive ELISA method with monoclonal antibodies detection was used for Protein Fingerprint biomarkers nordicPRO-C3^TM^, nordicPRO-C4^TM^, nordicPRO-C6^TM^, and nordicPRO-C18L^TM^ (reflecting formation of collagen III, IV, VI, and XVIII), as well as nordicC3M^TM^, nordicC4M^TM^, and nordicC6Ma3^TM^ (reflecting degradation of collagen III, IV, and VI).

Ninety-six well streptavidin plates coated with biotinylated synthetic peptide were dissolved in an optimized assay and incubated for 30 minutes at 20°C. A calibrator peptide amount of 20 µL or an appropriate dilution of analyte was added into the wells. This included 100 µL horseradish peroxidase conjugated with monoclonal antibodies directed against the specific sequence of interest and incubated for one or 20 hours at 4°C or 20°C, depending on the assay. One hundred µL tetramethylbenzidine (Kem‐En‐Tec cat. 4380H) was added and incubated for 15 minutes at 20°C in the dark. Sulfuric acid (100 µL, 1%) was used to stop reactions for measurements at 450 nm and with reference measurements performed at 650 nm. Samples were centrifuged at 300 rpm at incubation. Plates were washed five times after coating and sample incubation in a washing buffer (20 mmol/L Tris, 50 mmol/L NaCl, pH 7.2). A four-parametric fit model was used as calibration curves. For measurements below or above the lower- and upper limit of measurement range, values were recorded as the lowest or highest value within the detection range of the specific assay, respectively.

Notably, nordicPRO-C4^TM^ was not available in the validation cohort due to problems in the assay production pipeline while conducting biomarker measurements for the validation cohort.

# Supplementary results

## Linear regression

Considering that collagen degradation biomarkers followed the degree of PH and liver dysfunction, linear regression models were performed to identify the main determinants for collagen degradation products in the systemic circulation **(Supplementary Figure-8, Supplementary Table-2)**. Considering its major (patho-)physiological role in ECM degradation, TIMP-1 was included in regression models. Interestingly, HVPG, MELD, TIMP-1, and the fibrogenesis biomarkers PRO-C3/-C4 (entered separately into the models) were all significantly linked to C3M and C4M in univariate analysis, respectively. However, the collagen formation biomarkers remained the singular independent variables linked to their degradation products in multivariate analyses. PRO-C6 remained the only parameter linked to C6M in both uni- and multivariate analysis.

# Supplementary figures

**Supplementary Figure-1. Study flow chart.**


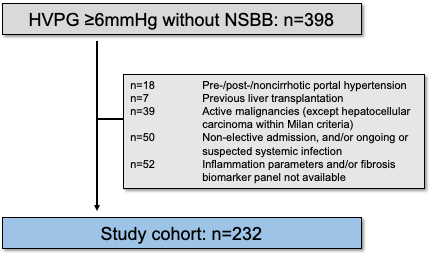


Abbreviations: (HVPG) hepatic venous pressure gradient; (NSBB) nonselective betablocker

**Supplementary Figure-2. ELF score and PRO-C18L levels in patients stratified by clinical disease stages, portal hypertension, and liver stiffness measurement.**

Figure legend: (ns) not significant; (*) p<0.05; (**) p<0.01; (***) p<0.001; (****) p<0.0001. HVPG groups: n=23 (12%) with 6-9 mmHg, n=124 (53%) with 10-19 mmHg, and n=80 (35%) with ≥20 mmHg. Statistical analysis: Group comparisons were performed by Wilcoxon Rank Sum test with Holm’s multiple comparisons adjustment. Abbreviations: (First/FurtherDec) first/further decompensation, (HVPG) Hepatic Venous Pressure Gradient, (ELF) Enhanced Liver Fibrosis, (PRO-C18L) Collagen Type XVIII formation marker, (LSM) liver stiffness measurement.

**Supplementary Figure-3. ECM biomarkers in patients stratified by LSM.**

Figure legend: LSM available in 202 (87%) of the patient cohort. LSM strata: n=78 (39%) with <25kPa, n=67 (33%) with 25-50 kPa, and n=57 (28%) with >50 kPa. (ns) not significant; (*) p<0.05; (**) p<0.01; (***) p<0.001; (****) p<0.0001. Statistical Analysis: Group comparisons were performed by Wilcoxon Rank Sum test with Holm’s multiple comparisons adjustment. Abbreviations: (LSM) liver stiffness measurement, (PRO-C3) Collagen Type III Propeptide, (PRO-C4) Collagen Type IV Propeptide, (PRO-C6) Collagen Type VI Propeptide, (C3M) Collagen Type III Metabolite, (C4M) Collagen Type IV Metabolite, (C6Ma3) Collagen Type VI Metabolite, (C3ratio) Collagen Type III Ratio, (C4ratio) Collagen Type IV Ratio, (C6ratio) Collagen Type VI Ratio.

**Supplementary Figure-4. Correlation between extracellular matrix (ECM) remodelling biomarkers and HVPG.**

Statistical analysis: Spearman’s correlation coefficient was calculated to assess the association between continuous variables. Abbreviations: (HVPG) Hepatic Venous Pressure Gradient, (ELF) Enhanced Liver Fibrosis, (TIMP1) Tissue Inhibitor of Metalloproteinases 1, (PRO-C3) Collagen Type III Propeptide, (PRO-C4) Collagen Type IV Propeptide, (PRO-C6) Collagen Type VI Propeptide, (PRO-C18L) Collagen Type XVIII formation marker, (C3M) Collagen Type III Metabolite, (C4M) Collagen Type IV Metabolite, (C6M) Collagen Type VI Metabolite, (C3ratio) Collagen Type III Ratio, (C4ratio) Collagen Type IV Ratio, (C6ratio) Collagen Type VI Ratio.

**Supplementary Figure-5. Correlation matrix of ECM remodeling biomarkers and parameters indicating systemic inflammation, portal hypertension, and liver dysfunction in the overall cohort.**


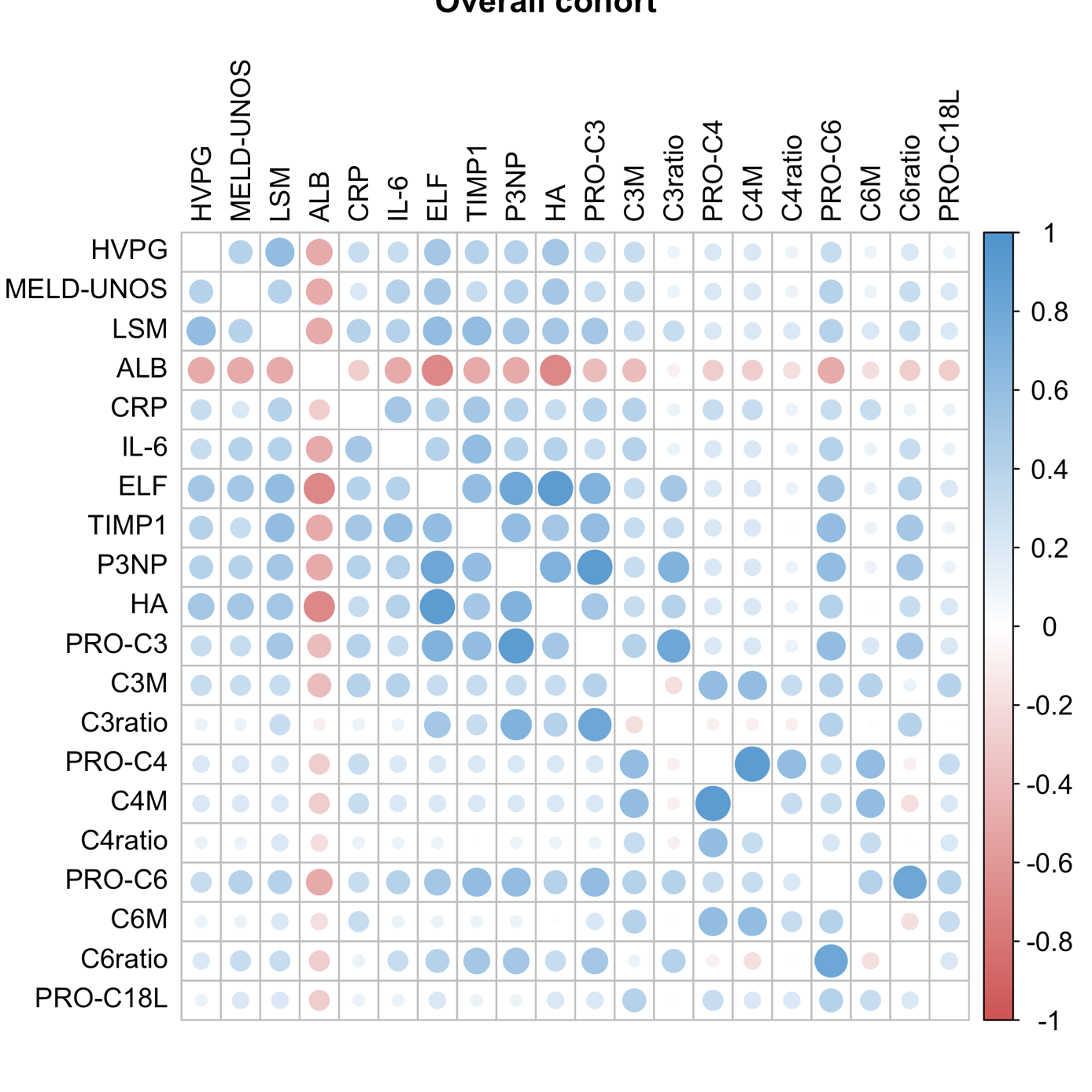


Statistical analysis: Spearman’s correlation coefficient was calculated to assess the association between continuous variables. Abbreviations: (HVPG) Hepatic Venous Pressure Gradient, (MELD) Model for End-Stage Liver Disease, (LSM) liver stiffness measurement; (IL-6) Interleukin-6, (ELF) Enhanced Liver Fibrosis score, (CRP) C-reactive protein, (TIMP1) Tissue Inhibitor of Metalloproteinases 1, (P3NP) Collagen Type III N-Terminal Propeptide, (HA) Hyaluronic Acid, (PRO-C3) Collagen Type III Propeptide, (PRO-C4) Collagen Type IV Propeptide, (PRO-C6) Collagen Type VI Propeptide, (PRO-C18L) Collagen Type XVIII formation marker, (C3M) Collagen Type III Metabolite, (C4M) Collagen Type IV Metabolite, (C6M) Collagen Type VI Metabolite, (C3ratio) Collagen Type III Ratio, (C4ratio) Collagen Type IV Ratio, (C6ratio) Collagen Type VI Ratio.

**Supplementary Figure-6. Histograms and Q-Q-Plots of variables included in linear regression models.**

Statistical analysis: Normality of distribution either without or with log-transformation of variables was assessed using the Shapiro-Wilk test. Abbreviations: (PRO-C3) Collagen Type III Propeptide, (PRO-C4) Collagen Type IV Propeptide, (C3M) Collagen Type III Metabolite, (C4M) Collagen Type IV Metabolite, (C3ratio) Collagen Type III Ratio, (C4ratio) Collagen Type IV Ratio.

**Supplementary Figure-7. Histograms and Q-Q-Plots of variables included in linear regression models.**

Statistical analysis: Normality of distribution either without or with log-transformation of variables was assessed using the Shapiro-Wilk test. Abbreviations: (HVPG) Hepatic Venous Pressure Gradient, (TIMP1) Tissue Inhibitor of Metalloproteinases 1, (PRO-C6) Collagen Type VI Propeptide, (PRO-C18L) Collagen Type XVIII formation marker, (C6M) Collagen Type VI Metabolite, (C6ratio) Collagen Type VI Ratio.

**Supplementary Figure-8. Uni- and multivariate linear regression model results.**

Statistical analysis: Uni- and multivariate linear regression model results are displayed by estimate values with 95% confidence intervals. Abbreviations: (HVPG) Hepatic Venous Pressure Gradient, (MELD) Model for End-Stage Liver Disease, (TIMP1) Tissue Inhibitor of Metalloproteinases 1, (PRO-C3) Collagen Type III Propeptide, (PRO-C4) Collagen Type IV Propeptide, (PRO-C6) Collagen Type VI Propeptide, (C3M) Collagen Type III Metabolite, (C4M) Collagen Type IV Metabolite, (C6M) Collagen Type VI Metabolite.

**Supplementary Figure-9. Incidence of liver-related events during the follow-up period: overall cohort stratified by terciles.**

Statistical Analysis: Kaplan-Meier curves indicating disease progression-free follow-up (composite endpoint: first/further decompensation, ACLF, and liver related death) were compared by log-rank test. Groups are defined by terciles (33^th^ and 66^th^ percentile) of the respective biomarkers. Abbreviations: (PRO-C3) Collagen Type III Propeptide, (PRO-C4) Collagen Type IV Propeptide, (PRO-C6) Collagen Type VI Propeptide, (C3M) Collagen Type III Metabolite, (C4M) Collagen Type IV Metabolite, (C6M) Collagen Type VI Metabolite, (C3ratio) Collagen Type III Ratio, (C4ratio) Collagen Type IV Ratio, (C6ratio) Collagen Type VI Ratio.

**Supplementary Figure-10. Incidence of first decompensation during the follow-up period: patients with compensated ACLD (cACLD) stratified by median biomarker levels.**

Statistical Analysis: Kaplan-Meier curves indicating disease progression-free follow-up (endpoint: first decompensation) were compared by log-rank test. Groups are defined as either below (“low”) or above (“high”) median biomarker levels. Abbreviations: (PRO-C3) Collagen Type III Propeptide, (PRO-C4) Collagen Type IV Propeptide, (PRO-C6) Collagen Type VI Propeptide, (C3M) Collagen Type III Metabolite, (C4M) Collagen Type IV Metabolite, (C6M) Collagen Type VI Metabolite, (C3ratio) Collagen Type III Ratio, (C4ratio) Collagen Type IV Ratio, (C6ratio) Collagen Type VI Ratio.

**Supplementary Figure-11. Incidence of first decompensation during the follow-up period: patients with compensated ACLD (cACLD) stratified by terciles.**

Statistical Analysis: Kaplan-Meier curves indicating disease progression-free follow-up (endpoint: first decompensation) were compared by log-rank test. Groups are defined by terciles (33^th^ and 66^th^ percentile) of the respective biomarkers. Abbreviations: (PRO-C3) Collagen Type III Propeptide, (PRO-C4) Collagen Type IV Propeptide, (PRO-C6) Collagen Type VI Propeptide, (C3M) Collagen Type III Metabolite, (C4M) Collagen Type IV Metabolite, (C6M) Collagen Type VI Metabolite, (C3ratio) Collagen Type III Ratio, (C4ratio) Collagen Type IV Ratio, (C6ratio) Collagen Type VI Ratio.

**Supplementary Figure-12. Incidence of liver-related events during the follow-up period: patients with decompensated ACLD (dACLD) stratified by median biomarker levels.**

Statistical Analysis: Kaplan-Meier curves indicating disease progression-free follow-up (composite endpoint: further decompensation, ACLF, and liver related death) were compared by log-rank test. Groups are defined as either below (“low”) or above (“high”) median biomarker levels. Abbreviations: (PRO-C3) Collagen Type III Propeptide, (PRO-C4) Collagen Type IV Propeptide, (PRO-C6) Collagen Type VI Propeptide, (C3M) Collagen Type III Metabolite, (C4M) Collagen Type IV Metabolite, (C6M) Collagen Type VI Metabolite, (C3ratio) Collagen Type III Ratio, (C4ratio) Collagen Type IV Ratio, (C6ratio) Collagen Type VI Ratio.

**Supplementary Figure-13. Incidence of liver-related events during the follow-up period: patients with decompensated ACLD (dACLD) stratified by terciles.**

Statistical Analysis: Kaplan-Meier curves indicating disease progression-free follow-up (composite endpoint: further decompensation, ACLF, and liver related death) were compared by log-rank test. Groups are defined by terciles (33^th^ and 66^th^ percentile) of the respective biomarkers. Abbreviations: (PRO-C3) Collagen Type III Propeptide, (PRO-C4) Collagen Type IV Propeptide, (PRO-C6) Collagen Type VI Propeptide, (C3M) Collagen Type III Metabolite, (C4M) Collagen Type IV Metabolite, (C6M) Collagen Type VI Metabolite, (C3ratio) Collagen Type III Ratio, (C4ratio) Collagen Type IV Ratio, (C6ratio) Collagen Type VI Ratio.

**Supplementary Figure-14. Incidence of liver-related events during the follow-up period: patients stratified by ELF score levels.**

Statistical Analysis: Kaplan-Meier curves indicating disease progression-free follow-up (overall cohort: first/further decompensation, ACLF, and liver related death; cACLD: first decompensation; dACLD: further decompensation, ACLF, and liver related death) were compared by log-rank test. (A) Groups are defined as either below (“low”) or above (“high”) median biomarker levels. (B) Groups are defined by terciles (33^th^ and 66^th^ percentile) of the respective biomarkers. Abbreviations: (ELF) Enhanced Liver Fibrosis score, (c/dACLD) compensated/decompensated advanced chronic liver disease.

**Supplementary Figure-15. Incidence of liver-related events during the follow-up period: patients stratified by PRO-C18L levels.**

Statistical Analysis: Kaplan-Meier curves indicating disease progression-free follow-up (overall cohort: first/further decompensation, ACLF, and liver related death; cACLD: first decompensation; dACLD: further decompensation, ACLF, and liver related death) were compared by log-rank test. (A) Groups are defined as either below (“low”) or above (“high”) median biomarker levels. (B) Groups are defined by terciles (33^th^ and 66^th^ percentile) of the respective biomarkers. Abbreviations: (PRO-C18L) Collagen Type XVIII formation marker, (c/dACLD) compensated/decompensated advanced chronic liver disease.

**Supplementary Figure-16. Time-dependent area-under-the-receiver operating characteristics (AUROC) for prediction of liver-related events: Collagen III, IV, and VI biomarkers.**

Statistical Analysis: Time-dependent area-under-the-receiver operating characteristics (AUROC) for prediction of liver-related events at 6, 12, 18, 24, 30, 36, 42, and 48 months. Abbreviations: (HVPG) hepatic venous pressure gradient, (MELD) Model for End-Stage Liver Disease, (ELF) enhanced liver fibrosis score, (PRO-C3) Collagen Type III Propeptide, (PRO-C4) Collagen Type IV Propeptide, (PRO-C6) Collagen Type VI Propeptide, (C3M) Collagen Type III Metabolite, (C4M) Collagen Type IV Metabolite, (C6M) Collagen Type VI Metabolite, (c/dACLD) compensated/decompensated advanced chronic liver disease, (LSM) liver stiffness measurement.

**Supplementary Figure-17. Time-dependent area-under-the-receiver operating characteristics (AUROC) for prediction of liver-related events: Collagen XVIII biomarker and ELF score.**

Statistical Analysis: Time-dependent area-under-the-receiver operating characteristics (AUROC) for prediction of liver-related events at 6, 12, 18, 24, 30, 36, 42, and 48 months. Abbreviations: (HVPG) hepatic venous pressure gradient, (MELD) Model for End-Stage Liver Disease, (ELF) enhanced liver fibrosis score, (PRO-C3) Collagen Type III Propeptide, (PRO-C4) Collagen Type IV Propeptide, (PRO-C6) Collagen Type VI Propeptide, (LSM) liver stiffness measurement.

**Supplementary Figure-18. Incidence of first decompensation during the follow-up period in the validation cohort: stratification by fibrosis biomarkers and HVPG.**

Statistical Analysis: Kaplan-Meier curves indicating disease progression-free follow-up (first decompensation) were compared by log-rank test. (A) Groups are defined as either below (“low”) or above (“high”) median biomarker levels. (B) Groups are defined by terciles (33^th^ and 66^th^ percentile). Abbreviations: (LSM) liver stiffness measurement, (cACLD) compensated/decompensated advanced chronic liver disease.

**Supplementary Figure-19. Time-dependent area-under-the-receiver operating characteristics (AUROC) for prediction of first decompensation in cACLD: validation cohort.**

Statistical Analysis: Time-dependent area-under-the-receiver operating characteristics (AUROC) for prediction of first hepatic decompensation at 12, 24, 36, and 48 months. Abbreviations: (HVPG) hepatic venous pressure gradient, (MELD) Model for End-Stage Liver Disease, (ELF) enhanced liver fibrosis score, (LSM) liver stiffness measurement, (PRO-C3) Collagen Type III Propeptide, (PRO-C6) Collagen Type VI Propeptide.

# Supplementary tables

**Supplementary Table-1. Correlation results in the overall cohort.**

|  | HVPG (mmHg) | UNOS-MELD (points) | LSM (kPa) | Albumin (mg/dL) | CRP (mg/dL) | IL-6 (pg/mL) | ELF score | TIMP1 (ng/mL) | P3NP (ng/mL) | HA (ng/mL) | PRO-C3 (ng/mL) | C3M (ng/mL) | C3ratio | PRO-C4 (ng/mL) | C4M (ng/mL) | C4ratio | PRO-C6 (ng/mL) | C6Ma3 (ng/mL) | C6ratio | PRO-C18L (ng/mL) |
| --- | --- | --- | --- | --- | --- | --- | --- | --- | --- | --- | --- | --- | --- | --- | --- | --- | --- | --- | --- | --- |
| HVPG (mmHg) |  | 0.393^***^ | 0.599^***^ | -0.456^***^ | 0.296^***^ | 0.324^***^ | 0.507^***^ | 0.374^***^ | 0.383^***^ | 0.498^***^ | 0.295^***^ | 0.323^***^ | 0.072 | 0.172^**^ | 0.190^**^ | 0.051 | 0.252^***^ | 0.095 | 0.174^**^ | 0.124 |
| UNOS-MELD (points) | 0.393^***^ |  | 0.370^***^ | -0.505^***^ | 0.215^**^ | 0.435^***^ | 0.534^***^ | 0.316^***^ | 0.401^***^ | 0.547^***^ | 0.281^***^ | 0.335^***^ | 0.112 | 0.233^***^ | 0.222^***^ | 0.149^*^ | 0.364^***^ | 0.075 | 0.312^***^ | 0.217^***^ |
| LSM (kPa) | 0.599^***^ | 0.370^***^ |  | -0.460^***^ | 0.391^***^ | 0.397^***^ | 0.570^***^ | 0.562^***^ | 0.534^***^ | 0.497^***^ | 0.504^***^ | 0.322^***^ | 0.309^***^ | 0.206^**^ | 0.167^*^ | 0.181^**^ | 0.445^***^ | 0.159^*^ | 0.328^***^ | 0.236^***^ |
| Albumin (mg/dL) | -0.456^***^ | -0.505^***^ | -0.460^***^ |  | -0.309^***^ | -0.538^***^ | -0.671^***^ | -0.523^***^ | -0.527^***^ | -0.659^***^ | -0.400^***^ | -0.422^***^ | -0.126 | -0.318^***^ | -0.281^***^ | -0.231^***^ | -0.469^***^ | -0.154^*^ | -0.348^***^ | -0.309^***^ |
| CRP (mg/dL) | 0.296^***^ | 0.215^**^ | 0.391^***^ | -0.309^***^ |  | 0.508^***^ | 0.372^***^ | 0.465^***^ | 0.393^***^ | 0.278^***^ | 0.351^***^ | 0.417^***^ | 0.100 | 0.319^***^ | 0.339^***^ | 0.121 | 0.321^***^ | 0.344^***^ | 0.087 | 0.099 |
| IL-6 (pg/mL) | 0.324^***^ | 0.435^***^ | 0.397^***^ | -0.538^***^ | 0.508^***^ |  | 0.433^***^ | 0.578^***^ | 0.376^***^ | 0.362^***^ | 0.325^***^ | 0.367^***^ | 0.067 | 0.207^**^ | 0.197^**^ | 0.122 | 0.433^***^ | 0.148^*^ | 0.318^***^ | 0.150^*^ |
| ELF score | 0.507^***^ | 0.534^***^ | 0.570^***^ | -0.671^***^ | 0.372^***^ | 0.433^***^ |  | 0.628^***^ | 0.847^***^ | 0.942^***^ | 0.729^***^ | 0.337^***^ | 0.529^***^ | 0.218^***^ | 0.207^**^ | 0.126 | 0.503^***^ | 0.059 | 0.418^***^ | 0.193^**^ |
| TIMP1 (ng/mL) | 0.374^***^ | 0.316^***^ | 0.562^***^ | -0.523^***^ | 0.465^***^ | 0.578^***^ | 0.628^***^ |  | 0.608^***^ | 0.455^***^ | 0.558^***^ | 0.348^***^ | 0.346^***^ | 0.197^**^ | 0.209^**^ | 0.044 | 0.572^***^ | 0.097 | 0.503^***^ | 0.132^*^ |
| P3NP (ng/mL) | 0.383^***^ | 0.401^***^ | 0.534^***^ | -0.527^***^ | 0.393^***^ | 0.376^***^ | 0.847^***^ | 0.608^***^ |  | 0.660^***^ | 0.880^***^ | 0.318^***^ | 0.694^***^ | 0.207^**^ | 0.194^**^ | 0.119 | 0.570^***^ | 0.100 | 0.485^***^ | 0.120 |
| HA (ng/mL) | 0.498^***^ | 0.547^***^ | 0.497^***^ | -0.659^***^ | 0.278^***^ | 0.362^***^ | 0.942^***^ | 0.455^***^ | 0.660^***^ |  | 0.547^***^ | 0.295^***^ | 0.374^***^ | 0.198^**^ | 0.175^**^ | 0.146^*^ | 0.383^***^ | 0.042 | 0.303^***^ | 0.217^***^ |
| PRO-C3 (ng/mL) | 0.295^***^ | 0.281^***^ | 0.504^***^ | -0.400^***^ | 0.351^***^ | 0.325^***^ | 0.729^***^ | 0.558^***^ | 0.880^***^ | 0.547^***^ |  | 0.369^***^ | 0.776^***^ | 0.237^***^ | 0.221^***^ | 0.142^*^ | 0.597^***^ | 0.181^**^ | 0.459^***^ | 0.202^**^ |
| C3M (ng/mL) | 0.323^***^ | 0.335^***^ | 0.322^***^ | -0.422^***^ | 0.417^***^ | 0.367^***^ | 0.337^***^ | 0.348^***^ | 0.318^***^ | 0.295^***^ | 0.369^***^ |  | -0.227^***^ | 0.633^***^ | 0.630^***^ | 0.338^***^ | 0.393^***^ | 0.388^***^ | 0.124 | 0.381^***^ |
| C3ratio | 0.072 | 0.112 | 0.309^***^ | -0.126 | 0.100 | 0.067 | 0.529^***^ | 0.346^***^ | 0.694^***^ | 0.374^***^ | 0.776^***^ | -0.227^***^ |  | -0.108 | -0.118 | -0.057 | 0.377^***^ | -0.018 | 0.388^***^ | -0.013 |
| PRO-C4 (ng/mL) | 0.172^**^ | 0.233^***^ | 0.206^**^ | -0.318^***^ | 0.319^***^ | 0.207^**^ | 0.218^***^ | 0.197^**^ | 0.207^**^ | 0.198^**^ | 0.237^***^ | 0.633^***^ | -0.108 |  | 0.894^***^ | 0.631^***^ | 0.301^***^ | 0.608^***^ | -0.103 | 0.282^***^ |
| C4M (ng/mL) | 0.190^**^ | 0.222^***^ | 0.167^*^ | -0.281^***^ | 0.339^***^ | 0.197^**^ | 0.207^**^ | 0.209^**^ | 0.194^**^ | 0.175^**^ | 0.221^***^ | 0.630^***^ | -0.118 | 0.894^***^ |  | 0.279^***^ | 0.260^***^ | 0.604^***^ | -0.154^*^ | 0.239^***^ |
| C4ratio | 0.051 | 0.149^*^ | 0.181^**^ | -0.231^***^ | 0.121 | 0.122 | 0.126 | 0.044 | 0.119 | 0.146^*^ | 0.142^*^ | 0.338^***^ | -0.057 | 0.631^***^ | 0.279^***^ |  | 0.206^**^ | 0.311^***^ | 0.019 | 0.238^***^ |
| PRO-C6 (ng/mL) | 0.252^***^ | 0.364^***^ | 0.445^***^ | -0.469^***^ | 0.321^***^ | 0.433^***^ | 0.503^***^ | 0.572^***^ | 0.570^***^ | 0.383^***^ | 0.597^***^ | 0.393^***^ | 0.377^***^ | 0.301^***^ | 0.260^***^ | 0.206^**^ |  | 0.360^***^ | 0.764^***^ | 0.403^***^ |
| C6Ma3 (ng/mL) | 0.095 | 0.075 | 0.159^*^ | -0.154^*^ | 0.344^***^ | 0.148^*^ | 0.059 | 0.097 | 0.100 | 0.042 | 0.181^**^ | 0.388^***^ | -0.018 | 0.608^***^ | 0.604^***^ | 0.311^***^ | 0.360^***^ |  | -0.248^***^ | 0.306^***^ |
| C6ratio | 0.174^**^ | 0.312^***^ | 0.328^***^ | -0.348^***^ | 0.087 | 0.318^***^ | 0.418^***^ | 0.503^***^ | 0.485^***^ | 0.303^***^ | 0.459^***^ | 0.124 | 0.388^***^ | -0.103 | -0.154^*^ | 0.019 | 0.764^***^ | -0.248^***^ |  | 0.230^***^ |
| PRO-C18L (ng/mL) | 0.124 | 0.217^***^ | 0.236^***^ | -0.309^***^ | 0.099 | 0.150^*^ | 0.193^**^ | 0.132^*^ | 0.120 | 0.217^***^ | 0.202^**^ | 0.381^***^ | -0.013 | 0.282^***^ | 0.239^***^ | 0.238^***^ | 0.403^***^ | 0.306^***^ | 0.230^***^ |  |
|  | | | | | | | | | | | | | | | | | | | | |

Figure legend: (*) p<0.05; (**) p<0.01; (***) p<0.001. Statistical Analysis: Spearman’s correlation coefficient was calculated to assess the association between continuous variables. Abbreviations: (c/dACLD) compensated/decompensated advanced chronic liver disease, (HVPG) Hepatic Venous Pressure Gradient, (MELD) Model for End-Stage Liver Disease, (LSM) liver stiffness measurement; (IL-6) Interleukin-6, (ELF) Enhanced Liver Fibrosis score, (LBP) lipopolysaccharide binding protein, (LSM) liver stiffness measurement, (TIMP1) Tissue Inhibitor of Metalloproteinases 1, (P3NP) Collagen Type III N-Terminal Propeptide, (HA) Hyaluronic Acid, (PRO-C3) Collagen Type III Propeptide, (PRO-C4) Collagen Type IV Propeptide, (PRO-C6) Collagen Type VI Propeptide, (PRO-C18L) Collagen Type XVIII formation marker, (C3M) Collagen Type III Metabolite, (C4M) Collagen Type IV Metabolite, (C6M) Collagen Type VI Metabolite, (C3ratio) Collagen Type III Ratio, (C4ratio) Collagen Type IV Ratio, (C6ratio) Collagen Type VI Ratio.

**Supplementary Table-2. Linear regression models assessing the independent relationship between patient characteristics and collagen turnover.**

| **C3M** | **Univariate** | | **Multivariate** | |
| --- | --- | --- | --- | --- |
| Age (years) | -0.00 | (-0.01, 0.00, p = 0.15) | -0.00 | (-0.01, 0.00, p = 0.36) |
| Sex (m) | -0.09 | (-0.19, 0.01, p = 0.08) | -0.04 | (-0.14, 0.05, p = 0.39) |
| HVPG (log) | 0.31 *** | (0.20, 0.43, p = 0.00) | 0.17 * | (0.04, 0.30, p = 0.01) |
| MELD (log) | 0.29 *** | (0.15, 0.43, p = 0.00) | 0.10 | (-0.04, 0.25, p = 0.17) |
| TIMP-1 (log) | 0.21 *** | (0.12, 0.31, p = 0.00) | 0.06 | (-0.04, 0.17, p = 0.25) |
| PRO-C3 (log) | 0.23 *** | (0.16, 0.31, p = 0.00) | 0.15 *** | (0.06, 0.24, p = 0.00) |
| **C4M** | **Univariate** | | **Multivariate** | |
| Age (years) | 0.00 | (-0.00, 0.00, p = 0.93) | -0.00 | (-0.00, 0.00, p = 0.35) |
| Sex (m) | 0.05 | (-0.06, 0.16, p = 0.39) | 0.01 | (-0.05, 0.07, p = 0.77) |
| HVPG (log) | 0.18 ** | (0.05, 0.31, p = 0.01) | 0.03 | (-0.06, 0.11, p = 0.54) |
| MELD (log) | 0.21 ** | (0.06, 0.37, p = 0.01) | 0.04 | (-0.06, 0.13, p = 0.46) |
| TIMP-1 (log) | 0.13 * | (0.02, 0.23, p = 0.02) | 0.04 | (-0.03, 0.10, p = 0.25) |
| PRO-C4 (log) | 0.62 *** | (0.57, 0.67, p = 0.00) | 0.61 *** | (0.56, 0.67, p = 0.00) |
| **C6M** | **Univariate** | | **Multivariate** | |
| Age (years) | 0.00 | (-0.00, 0.00, p = 0.93) | -0.00 | (-0.00, 0.00, p = 0.88) |
| Sex (m) | -0.00 | (-0.09, 0.09, p = 0.93) | -0.01 | (-0.10, 0.08, p = 0.79) |
| HVPG (log) | 0.08 | (-0.03, 0.19, p = 0.16) | 0.05 | (-0.07, 0.17, p = 0.43) |
| MELD (log) | 0.08 | (-0.05, 0.21, p = 0.24) | -0.08 | (-0.22, 0.07, p = 0.30) |
| TIMP-1 (log) | 0.04 | (-0.04, 0.13, p = 0.33) | -0.05 | (-0.14, 0.04, p = 0.28) |
| PRO-C6 (log) | 0.23 *** | (0.15, 0.31, p = 0.00) | 0.26 *** | (0.17, 0.35, p = 0.00) |

Figure legend: (*) p<0.05; (**) p<0.01; (***) p<0.001. Statistical Analysis: Uni- and multivariate linear regression model results are displayed by estimate values with 95% confidence intervals. Abbreviations: (HVPG) Hepatic Venous Pressure Gradient, (MELD) Model for End-Stage Liver Disease, (TIMP1) Tissue Inhibitor of Metalloproteinases 1, (PRO-C3) Collagen Type III Propeptide, (PRO-C4) Collagen Type IV Propeptide, (PRO-C6) Collagen Type VI Propeptide, (C3M) Collagen Type III Metabolite, (C4M) Collagen Type IV Metabolite, (C6M) Collagen Type VI Metabolite, (C3ratio) Collagen Type III Ratio, (C4ratio) Collagen Type IV Ratio, (C6ratio) Collagen Type VI Ratio.

**Supplementary Table-3. Correlation results in patients with compensated ACLD (cACLD).**

|  | HVPG (mmHg) | UNOS-MELD (points) | LSM (kPa) | Albumin (mg/dL) | CRP (mg/dL) | IL-6 (pg/mL) | ELF score | TIMP1 (ng/mL) | P3NP (ng/mL) | HA (ng/mL) | PRO-C3 (ng/mL) | C3M (ng/mL) | C3ratio | PRO-C4 (ng/mL) | C4M (ng/mL) | C4ratio | PRO-C6 (ng/mL) | C6Ma3 (ng/mL) | C6ratio | PRO-C18L (ng/mL) |
| --- | --- | --- | --- | --- | --- | --- | --- | --- | --- | --- | --- | --- | --- | --- | --- | --- | --- | --- | --- | --- |
| HVPG (mmHg) |  | 0.348^***^ | 0.496^***^ | -0.441^***^ | 0.225^*^ | 0.163 | 0.609^***^ | 0.348^***^ | 0.430^***^ | 0.607^***^ | 0.443^***^ | 0.264^**^ | 0.315^**^ | 0.199^*^ | 0.230^*^ | -0.005 | 0.191 | 0.075 | 0.114 | 0.096 |
| UNOS-MELD (points) | 0.348^***^ |  | 0.285^**^ | -0.365^***^ | 0.091 | 0.375^***^ | 0.476^***^ | 0.303^**^ | 0.303^**^ | 0.509^***^ | 0.172 | 0.237^*^ | 0.082 | 0.167 | 0.180 | 0.088 | 0.212^*^ | -0.018 | 0.229^*^ | 0.088 |
| LSM (kPa) | 0.496^***^ | 0.285^**^ |  | -0.255^*^ | 0.223^*^ | 0.228^*^ | 0.473^***^ | 0.453^***^ | 0.411^***^ | 0.401^***^ | 0.449^***^ | 0.191 | 0.369^***^ | 0.090 | 0.011 | 0.140 | 0.369^***^ | 0.008 | 0.324^**^ | 0.061 |
| Albumin (mg/dL) | -0.441^***^ | -0.365^***^ | -0.255^*^ |  | -0.223^*^ | -0.393^***^ | -0.619^***^ | -0.460^***^ | -0.476^***^ | -0.608^***^ | -0.412^***^ | -0.491^***^ | -0.157 | -0.409^***^ | -0.369^***^ | -0.216^*^ | -0.331^***^ | -0.155 | -0.150 | -0.142 |
| CRP (mg/dL) | 0.225^*^ | 0.091 | 0.223^*^ | -0.223^*^ |  | 0.454^***^ | 0.338^***^ | 0.351^***^ | 0.304^**^ | 0.282^**^ | 0.332^***^ | 0.293^**^ | 0.175 | 0.267^**^ | 0.288^**^ | 0.082 | 0.229^*^ | 0.222^*^ | 0.050 | 0.052 |
| IL-6 (pg/mL) | 0.163 | 0.375^***^ | 0.228^*^ | -0.393^***^ | 0.454^***^ |  | 0.346^***^ | 0.430^***^ | 0.314^**^ | 0.292^**^ | 0.361^***^ | 0.274^**^ | 0.176 | 0.186 | 0.212^*^ | 0.055 | 0.318^**^ | 0.004 | 0.281^**^ | -0.049 |
| ELF score | 0.609^***^ | 0.476^***^ | 0.473^***^ | -0.619^***^ | 0.338^***^ | 0.346^***^ |  | 0.590^***^ | 0.845^***^ | 0.928^***^ | 0.719^***^ | 0.317^**^ | 0.577^***^ | 0.244^*^ | 0.268^**^ | 0.066 | 0.380^***^ | -0.010 | 0.283^**^ | 0.007 |
| TIMP1 (ng/mL) | 0.348^***^ | 0.303^**^ | 0.453^***^ | -0.460^***^ | 0.351^***^ | 0.430^***^ | 0.590^***^ |  | 0.583^***^ | 0.395^***^ | 0.609^***^ | 0.360^***^ | 0.424^***^ | 0.225^*^ | 0.230^*^ | -0.004 | 0.537^***^ | 0.051 | 0.447^***^ | 0.065 |
| P3NP (ng/mL) | 0.430^***^ | 0.303^**^ | 0.411^***^ | -0.476^***^ | 0.304^**^ | 0.314^**^ | 0.845^***^ | 0.583^***^ |  | 0.629^***^ | 0.842^***^ | 0.259^**^ | 0.729^***^ | 0.224^*^ | 0.234^*^ | 0.090 | 0.417^***^ | 0.003 | 0.331^***^ | -0.100 |
| HA (ng/mL) | 0.607^***^ | 0.509^***^ | 0.401^***^ | -0.608^***^ | 0.282^**^ | 0.292^**^ | 0.928^***^ | 0.395^***^ | 0.629^***^ |  | 0.530^***^ | 0.305^**^ | 0.395^***^ | 0.224^*^ | 0.244^*^ | 0.087 | 0.263^**^ | 0.002 | 0.172 | 0.047 |
| PRO-C3 (ng/mL) | 0.443^***^ | 0.172 | 0.449^***^ | -0.412^***^ | 0.332^***^ | 0.361^***^ | 0.719^***^ | 0.609^***^ | 0.842^***^ | 0.530^***^ |  | 0.374^***^ | 0.813^***^ | 0.228^*^ | 0.253^*^ | 0.088 | 0.579^***^ | 0.136 | 0.429^***^ | 0.029 |
| C3M (ng/mL) | 0.264^**^ | 0.237^*^ | 0.191 | -0.491^***^ | 0.293^**^ | 0.274^**^ | 0.317^**^ | 0.360^***^ | 0.259^**^ | 0.305^**^ | 0.374^***^ |  | -0.173 | 0.565^***^ | 0.631^***^ | 0.184 | 0.361^***^ | 0.365^***^ | 0.024 | 0.396^***^ |
| C3ratio | 0.315^**^ | 0.082 | 0.369^***^ | -0.157 | 0.175 | 0.176 | 0.577^***^ | 0.424^***^ | 0.729^***^ | 0.395^***^ | 0.813^***^ | -0.173 |  | -0.079 | -0.073 | -0.037 | 0.384^***^ | -0.052 | 0.434^***^ | -0.193 |
| PRO-C4 (ng/mL) | 0.199^*^ | 0.167 | 0.090 | -0.409^***^ | 0.267^**^ | 0.186 | 0.244^*^ | 0.225^*^ | 0.224^*^ | 0.224^*^ | 0.228^*^ | 0.565^***^ | -0.079 |  | 0.863^***^ | 0.542^***^ | 0.335^***^ | 0.589^***^ | -0.163 | 0.260^**^ |
| C4M (ng/mL) | 0.230^*^ | 0.180 | 0.011 | -0.369^***^ | 0.288^**^ | 0.212^*^ | 0.268^**^ | 0.230^*^ | 0.234^*^ | 0.244^*^ | 0.253^*^ | 0.631^***^ | -0.073 | 0.863^***^ |  | 0.133 | 0.259^**^ | 0.598^***^ | -0.236^*^ | 0.257^**^ |
| C4ratio | -0.005 | 0.088 | 0.140 | -0.216^*^ | 0.082 | 0.055 | 0.066 | -0.004 | 0.090 | 0.087 | 0.088 | 0.184 | -0.037 | 0.542^***^ | 0.133 |  | 0.245^*^ | 0.253^*^ | 0.013 | 0.185 |
| PRO-C6 (ng/mL) | 0.191 | 0.212^*^ | 0.369^***^ | -0.331^***^ | 0.229^*^ | 0.318^**^ | 0.380^***^ | 0.537^***^ | 0.417^***^ | 0.263^**^ | 0.579^***^ | 0.361^***^ | 0.384^***^ | 0.335^***^ | 0.259^**^ | 0.245^*^ |  | 0.360^***^ | 0.664^***^ | 0.357^***^ |
| C6Ma3 (ng/mL) | 0.075 | -0.018 | 0.008 | -0.155 | 0.222^*^ | 0.004 | -0.010 | 0.051 | 0.003 | 0.002 | 0.136 | 0.365^***^ | -0.052 | 0.589^***^ | 0.598^***^ | 0.253^*^ | 0.360^***^ |  | -0.346^***^ | 0.300^**^ |
| C6ratio | 0.114 | 0.229^*^ | 0.324^**^ | -0.150 | 0.050 | 0.281^**^ | 0.283^**^ | 0.447^***^ | 0.331^***^ | 0.172 | 0.429^***^ | 0.024 | 0.434^***^ | -0.163 | -0.236^*^ | 0.013 | 0.664^***^ | -0.346^***^ |  | 0.132 |
| PRO-C18L (ng/mL) | 0.096 | 0.088 | 0.061 | -0.142 | 0.052 | -0.049 | 0.007 | 0.065 | -0.100 | 0.047 | 0.029 | 0.396^***^ | -0.193 | 0.260^**^ | 0.257^**^ | 0.185 | 0.357^***^ | 0.300^**^ | 0.132 |  |
|  | | | | | | | | | | | | | | | | | | | | |

Figure legend: (*) p<0.05; (**) p<0.01; (***) p<0.001. Statistical Analysis: Spearman’s correlation coefficient was calculated to assess the association between continuous variables. Abbreviations: (c/dACLD) compensated/decompensated advanced chronic liver disease, (HVPG) Hepatic Venous Pressure Gradient, (MELD) Model for End-Stage Liver Disease, (IL-6) Interleukin-6, (ELF) Enhanced Liver Fibrosis score, (LBP) lipopolysaccharide binding protein, (LSM) liver stiffness measurement, (TIMP1) Tissue Inhibitor of Metalloproteinases 1, (P3NP) Collagen Type III N-Terminal Propeptide, (HA) Hyaluronic Acid, (PRO-C3) Collagen Type III Propeptide, (PRO-C4) Collagen Type IV Propeptide, (PRO-C6) Collagen Type VI Propeptide, (PRO-C18L) Collagen Type XVIII formation marker, (C3M) Collagen Type III Metabolite, (C4M) Collagen Type IV Metabolite, (C6M) Collagen Type VI Metabolite, (C3ratio) Collagen Type III Ratio, (C4ratio) Collagen Type IV Ratio, (C6ratio) Collagen Type VI Ratio.

**Supplementary Table-4. Correlation results in patients with decompensated ACLD (dACLD).**

|  | HVPG (mmHg) | UNOS-MELD (points) | LSM (kPa) | Albumin (mg/dL) | CRP (mg/dL) | IL-6 (pg/mL) | ELF score | TIMP1 (ng/mL) | P3NP (ng/mL) | HA (ng/mL) | PRO-C3 (ng/mL) | C3M (ng/mL) | C3ratio | PRO-C4 (ng/mL) | C4M (ng/mL) | C4ratio | PRO-C6 (ng/mL) | C6Ma3 (ng/mL) | C6ratio | PRO-C18L (ng/mL) |
| --- | --- | --- | --- | --- | --- | --- | --- | --- | --- | --- | --- | --- | --- | --- | --- | --- | --- | --- | --- | --- |
| HVPG (mmHg) |  | 0.250^**^ | 0.530^***^ | -0.297^***^ | 0.219^*^ | 0.308^***^ | 0.278^**^ | 0.280^**^ | 0.109 | 0.309^***^ | 0.050 | 0.280^**^ | -0.169 | 0.008 | 0.047 | -0.046 | 0.055 | 0.005 | 0.026 | 0.031 |
| UNOS-MELD (points) | 0.250^**^ |  | 0.260^**^ | -0.427^***^ | 0.172^*^ | 0.372^***^ | 0.469^***^ | 0.209^*^ | 0.318^***^ | 0.492^***^ | 0.246^**^ | 0.277^**^ | 0.110 | 0.174^*^ | 0.142 | 0.112 | 0.309^***^ | 0.069 | 0.235^**^ | 0.257^**^ |
| LSM (kPa) | 0.530^***^ | 0.260^**^ |  | -0.400^***^ | 0.420^***^ | 0.432^***^ | 0.490^***^ | 0.575^***^ | 0.433^***^ | 0.405^***^ | 0.442^***^ | 0.291^**^ | 0.230^*^ | 0.126 | 0.123 | 0.103 | 0.367^***^ | 0.183 | 0.241^*^ | 0.280^**^ |
| Albumin (mg/dL) | -0.297^***^ | -0.427^***^ | -0.400^***^ |  | -0.217^*^ | -0.498^***^ | -0.589^***^ | -0.452^***^ | -0.380^***^ | -0.598^***^ | -0.260^**^ | -0.289^***^ | -0.030 | -0.110 | -0.091 | -0.086 | -0.351^***^ | -0.028 | -0.327^***^ | -0.403^***^ |
| CRP (mg/dL) | 0.219^*^ | 0.172^*^ | 0.420^***^ | -0.217^*^ |  | 0.469^***^ | 0.272^**^ | 0.486^***^ | 0.328^***^ | 0.141 | 0.289^***^ | 0.441^***^ | 0.015 | 0.294^***^ | 0.335^***^ | 0.081 | 0.305^***^ | 0.414^***^ | 0.017 | 0.070 |
| IL-6 (pg/mL) | 0.308^***^ | 0.372^***^ | 0.432^***^ | -0.498^***^ | 0.469^***^ |  | 0.372^***^ | 0.625^***^ | 0.282^**^ | 0.290^***^ | 0.201^*^ | 0.315^***^ | -0.032 | 0.098 | 0.099 | 0.074 | 0.400^***^ | 0.166 | 0.267^**^ | 0.222^*^ |
| ELF score | 0.278^**^ | 0.469^***^ | 0.490^***^ | -0.589^***^ | 0.272^**^ | 0.372^***^ |  | 0.583^***^ | 0.799^***^ | 0.927^***^ | 0.695^***^ | 0.255^**^ | 0.507^***^ | 0.080 | 0.032 | 0.077 | 0.482^***^ | 0.005 | 0.424^***^ | 0.276^**^ |
| TIMP1 (ng/mL) | 0.280^**^ | 0.209^*^ | 0.575^***^ | -0.452^***^ | 0.486^***^ | 0.625^***^ | 0.583^***^ |  | 0.536^***^ | 0.387^***^ | 0.466^***^ | 0.279^**^ | 0.272^**^ | 0.102 | 0.130 | -0.000 | 0.532^***^ | 0.064 | 0.474^***^ | 0.112 |
| P3NP (ng/mL) | 0.109 | 0.318^***^ | 0.433^***^ | -0.380^***^ | 0.328^***^ | 0.282^**^ | 0.799^***^ | 0.536^***^ |  | 0.575^***^ | 0.893^***^ | 0.245^**^ | 0.701^***^ | 0.089 | 0.047 | 0.080 | 0.578^***^ | 0.072 | 0.517^***^ | 0.181^*^ |
| HA (ng/mL) | 0.309^***^ | 0.492^***^ | 0.405^***^ | -0.598^***^ | 0.141 | 0.290^***^ | 0.927^***^ | 0.387^***^ | 0.575^***^ |  | 0.471^***^ | 0.194^*^ | 0.328^***^ | 0.060 | -0.006 | 0.101 | 0.332^***^ | -0.021 | 0.279^**^ | 0.321^***^ |
| PRO-C3 (ng/mL) | 0.050 | 0.246^**^ | 0.442^***^ | -0.260^**^ | 0.289^***^ | 0.201^*^ | 0.695^***^ | 0.466^***^ | 0.893^***^ | 0.471^***^ |  | 0.294^***^ | 0.767^***^ | 0.170 | 0.117 | 0.140 | 0.558^***^ | 0.151 | 0.432^***^ | 0.270^**^ |
| C3M (ng/mL) | 0.280^**^ | 0.277^**^ | 0.291^**^ | -0.289^***^ | 0.441^***^ | 0.315^***^ | 0.255^**^ | 0.279^**^ | 0.245^**^ | 0.194^*^ | 0.294^***^ |  | -0.305^***^ | 0.642^***^ | 0.612^***^ | 0.383^***^ | 0.352^***^ | 0.366^***^ | 0.126 | 0.334^***^ |
| C3ratio | -0.169 | 0.110 | 0.230^*^ | -0.030 | 0.015 | -0.032 | 0.507^***^ | 0.272^**^ | 0.701^***^ | 0.328^***^ | 0.767^***^ | -0.305^***^ |  | -0.157 | -0.190^*^ | -0.072 | 0.354^***^ | -0.011 | 0.328^***^ | 0.091 |
| PRO-C4 (ng/mL) | 0.008 | 0.174^*^ | 0.126 | -0.110 | 0.294^***^ | 0.098 | 0.080 | 0.102 | 0.089 | 0.060 | 0.170 | 0.642^***^ | -0.157 |  | 0.904^***^ | 0.672^***^ | 0.205^*^ | 0.604^***^ | -0.164 | 0.274^**^ |
| C4M (ng/mL) | 0.047 | 0.142 | 0.123 | -0.091 | 0.335^***^ | 0.099 | 0.032 | 0.130 | 0.047 | -0.006 | 0.117 | 0.612^***^ | -0.190^*^ | 0.904^***^ |  | 0.344^***^ | 0.180^*^ | 0.595^***^ | -0.196^*^ | 0.178^*^ |
| C4ratio | -0.046 | 0.112 | 0.103 | -0.086 | 0.081 | 0.074 | 0.077 | -0.000 | 0.080 | 0.101 | 0.140 | 0.383^***^ | -0.072 | 0.672^***^ | 0.344^***^ |  | 0.110 | 0.326^***^ | -0.060 | 0.266^**^ |
| PRO-C6 (ng/mL) | 0.055 | 0.309^***^ | 0.367^***^ | -0.351^***^ | 0.305^***^ | 0.400^***^ | 0.482^***^ | 0.532^***^ | 0.578^***^ | 0.332^***^ | 0.558^***^ | 0.352^***^ | 0.354^***^ | 0.205^*^ | 0.180^*^ | 0.110 |  | 0.296^***^ | 0.777^***^ | 0.392^***^ |
| C6Ma3 (ng/mL) | 0.005 | 0.069 | 0.183 | -0.028 | 0.414^***^ | 0.166 | 0.005 | 0.064 | 0.072 | -0.021 | 0.151 | 0.366^***^ | -0.011 | 0.604^***^ | 0.595^***^ | 0.326^***^ | 0.296^***^ |  | -0.295^***^ | 0.284^***^ |
| C6ratio | 0.026 | 0.235^**^ | 0.241^*^ | -0.327^***^ | 0.017 | 0.267^**^ | 0.424^***^ | 0.474^***^ | 0.517^***^ | 0.279^**^ | 0.432^***^ | 0.126 | 0.328^***^ | -0.164 | -0.196^*^ | -0.060 | 0.777^***^ | -0.295^***^ |  | 0.210^*^ |
| PRO-C18L (ng/mL) | 0.031 | 0.257^**^ | 0.280^**^ | -0.403^***^ | 0.070 | 0.222^*^ | 0.276^**^ | 0.112 | 0.181^*^ | 0.321^***^ | 0.270^**^ | 0.334^***^ | 0.091 | 0.274^**^ | 0.178^*^ | 0.266^**^ | 0.392^***^ | 0.284^***^ | 0.210^*^ |  |
|  | | | | | | | | | | | | | | | | | | | | |

Figure legend: (*) p<0.05; (**) p<0.01; (***) p<0.001. Statistical Analysis: Spearman’s correlation coefficient was calculated to assess the association between continuous variables. Abbreviations: (c/dACLD) compensated/decompensated advanced chronic liver disease, (HVPG) Hepatic Venous Pressure Gradient, (MELD) Model for End-Stage Liver Disease, (IL-6) Interleukin-6, (ELF) Enhanced Liver Fibrosis score, (LBP) lipopolysaccharide binding protein, (LSM) liver stiffness measurement, (TIMP1) Tissue Inhibitor of Metalloproteinases 1, (P3NP) Collagen Type III N-Terminal Propeptide, (HA) Hyaluronic Acid, (PRO-C3) Collagen Type III Propeptide, (PRO-C4) Collagen Type IV Propeptide, (PRO-C6) Collagen Type VI Propeptide, (PRO-C18L) Collagen Type XVIII formation marker, (C3M) Collagen Type III Metabolite, (C4M) Collagen Type IV Metabolite, (C6M) Collagen Type VI Metabolite, (C3ratio) Collagen Type III Ratio, (C4ratio) Collagen Type IV Ratio, (C6ratio) Collagen Type VI Ratio.

**Supplementary Table-5. Cox regression models for the prediction of disease progression in patients with compensated ACLD (cACLD).**

| **cACLD**  **C3-biomarkers** | **Univariable** | | **Multivariable (first model)** | | **Multivariable (last model)** | |
| --- | --- | --- | --- | --- | --- | --- |
|  | **HR (95%CI)** | **p-value** | **aHR (95%CI)** | **p-value** | **aHR (95%CI)** | **p-value** |
| Age (per year) | 1.00 (0.97-1.03) | **0.788** | 1.00 (0.97-1.03) | 0.907 | - | - |
| HVPG (mmHg) | 1.12 (1.04-1.20) | **0.003** | 1.07 (0.98-1.16) | 0.154 | 1.09 (1.00-1.19) | **0.042** |
| MELD (points) | 1.15 (1.04-1.29) | **0.010** | 1.04 (0.90-1.21) | 0.579 | - | - |
| Albumin (g/L) | 0.81 (0.74-0.90) | **<0.001** | 0.86 (0.76-0.98) | **0.021** | 0.82 (0.74-0.92) | **<0.001** |
| PRO-C3 (ng/mL) | 1.06 (1.02-1.09) | **<0.001** | 1.02 (0.99-1.06) | 0.178 | - | - |
| C3M (ng/mL) | 1.09 (1.01-1.18) | **0.021** | 1.03 (0.93-1.14) | 0.587 | - | - |
| C3ratio | 1.88 (1.15-3.06) | **0.012** | - | - | - | - |
| **C4-biomarkers** | **HR (95%CI)** | **p-value** | **aHR (95%CI)** | **p-value** | **aHR (95%CI)** | **p-value** |
| Age (per year) | 1.00 (0.97-1.03) | **0.788** | 0.99 (0.95-1.02) | 0.506 | - | - |
| HVPG (mmHg) | 1.12 (1.04-1.20) | **0.003** | 1.07 (0.98-1.16) | 0.136 | 1.09 (1.00-1.19) | **0.042** |
| MELD (points) | 1.15 (1.04-1.29) | **0.010** | 1.09 (0.94-1.26) | 0.256 | - | - |
| Albumin (g/L) | 0.81 (0.74-0.90) | **<0.001** | 0.86 (0.76-0.97) | 0.017 | 0.82 (0.74-0.92) | **<0.001** |
| PRO-C4 (per 100 ng/mL) | 1.22 (1.11-1.35) | **<0.001** | 1.22 (0.84-1.76) | 0.299 | - | - |
| C4M (ng/mL) | 1.04 (1.02-1.06) | **<0.001** | 0.99 (0.93-1.07) | 0.939 | - | - |
| C4ratio | 1.33 (1.12-1.57) | **<0.001** | - | - | - | - |
| **C6-biomarkers** | **HR (95%CI)** | **p-value** | **aHR (95%CI)** | **p-value** | **aHR (95%CI)** | **p-value** |
| Age (per year) | 1.00 (0.97-1.03) | **0.788** | 0.99 (0.96-1.02) | 0.769 | - | - |
| HVPG (mmHg) | 1.12 (1.04-1.20) | **0.003** | 1.07 (0.98-1.18) | 0.101 | 1.08 (0.99-1.18) | 0.081 |
| MELD (points) | 1.15 (1.04-1.29) | **0.010** | 1.06 (0.92-1.23) | 0.420 | - | - |
| Albumin (g/L) | 0.81 (0.74-0.90) | **<0.001** | 0.86 (0.76-0.97) | 0.010 | 0.83 (0.74-0.93) | **0.001** |
| PRO-C6 (ng/mL) | 1.03 (0.99-1.06) | 0.111 | 1.01 (0.97-1.06) | 0.631 | - | - |
| C6M (ng/mL) | 37.3 (2.20-632) | **0.012** | 15.9 (0.47-535) | 0.122 | 17.43 (0.77-396) | 0.073 |
| C6ratio | 1.00 (0.99-1.02) | 0.495 | - | - | - | - |
| **ELF score** | **HR (95%CI)** | **p-value** | **aHR (95%CI)** | **p-value** | **aHR (95%CI)** | **p-value** |
| Age (per year) | 1.00 (0.97-1.03) | **0.788** | 0.99 (0.96-1.03) | 0.681 | - | - |
| HVPG (mmHg) | 1.12 (1.04-1.20) | **0.003** | 1.08 (0.98-1.17) | 0.144 | 1.09 (1.00-1.19) | **0.042** |
| MELD (points) | 1.15 (1.04-1.29) | **0.010** | 1.05 (0.91-1.21) | 0.498 | - | - |
| Albumin (g/L) | 0.81 (0.74-0.90) | **<0.001** | 0.88 (0.76-1.01) | 0.065 | 0.82 (0.74-0.92) | **<0.001** |
| ELF score | 1.75 (1.32-2.30) | **<0.001** | 1.26 (0.83-1.91) | 0.275 | - | - |
| **PRO-C18L** | **HR (95%CI)** | **p-value** | **aHR (95%CI)** | **p-value** | **aHR (95%CI)** | **p-value** |
| Age (per year) | 1.00 (0.97-1.03) | **0.788** | 0.99 (0.97 - 1.03) | 0.843 | - | - |
| HVPG (mmHg) | 1.12 (1.04-1.20) | **0.003** | 1.09 (1.00 - 1.18) | 0.058 | 1.09 (1.00-1.19) | **0.042** |
| MELD (points) | 1.15 (1.04-1.29) | **0.010** | 1.07 (0.93 - 1.22) | 0.356 | - | - |
| Albumin (g/L) | 0.81 (0.74-0.90) | **<0.001** | 0.84 (0.75 - 0.95) | **0.005** | 0.82 (0.74-0.92) | **<0.001** |
| PRO-C18L (ng/mL) | 0.99 (0.91-1.07) | 0.798 | 1.00 (0.90 - 1.11) | 0.981 | - | - |

Statistical Analysis: Uni- and multivariable Cox regression analysis of factors associated with first hepatic decompensation (n=22 events). Abbreviations: (aHR) adjusted hazard ratio, (HVPG) Hepatic Venous Pressure Gradient, (MELD) Model for End-Stage Liver Disease, (PRO-C3) Collagen Type III Propeptide, (PRO-C4) Collagen Type IV Propeptide, (PRO-C6) Collagen Type VI Propeptide, (C3M) Collagen Type III Metabolite, (C4M) Collagen Type IV Metabolite, (C6M) Collagen Type VI Metabolite, (C3ratio) Collagen Type III Ratio, (C4ratio) Collagen Type IV Ratio, (C6ratio) Collagen Type VI Ratio, (ELF) enhanced liver fibrosis score, (PRO-C18L) Collagen Type XVIII formation marker.

**Supplementary Table-6. Cox regression models for the prediction of disease progression in patients with decompensated ACLD (dACLD).**

| **dACLD**  **C3-biomarkers** | **Univariable** | | **Multivariable (first model)** | | **Multivariable (last model)** | |
| --- | --- | --- | --- | --- | --- | --- |
|  | **HR (95%CI)** | **p-value** | **aHR (95%CI)** | **p-value** | **aHR (95%CI)** | **p-value** |
| Age (per year) | 1.01 (0.99-1.04) | 0.220 | 1.01 (0.99-1.04) | 0.282 | - | - |
| HVPG (mmHg) | 1.05 (1.01-1.10) | **0.012** | 1.05 (1.01-1.10) | **0.012** | 1.05 (1.01-1.09) | **0.018** |
| MELD (points) | 1.02 (0.97-1.08) | 0.346 | 0.99 (0.92-1.06) | 0.769 | - | - |
| Albumin (g/L) | 0.94 (0.89-0.98) | **0.007** | 0.92 (0.87-0.98) | **0.007** | 0.93 (0.88-0.97) | **0.003** |
| PRO-C3 (ng/mL) | 0.99 (0.97-1.00) | **0.049** | 0.99 (0.97-1.00) | 0.069 | 0.98 (0.97-1.00) | **0.035** |
| C3M (ng/mL) | 0.99 (0.97-1.01) | 0.300 | 0.98 (0.96-1.00) | 0.072 | 0.98 (0.96-1.00) | 0.094 |
| C3ratio | 0.79 (0.62-1.00) | 0.053 | - | - | - | - |
| **C4-biomarkers** | **HR (95%CI)** | **p-value** | **aHR (95%CI)** | **p-value** | **aHR (95%CI)** | **p-value** |
| Age (per year) | 1.01 (0.99-1.04) | 0.220 | 1.01 (0.99-1.03) | 0.198 | - | - |
| HVPG (mmHg) | 1.05 (1.01-1.10) | 0.012 | 1.04 (0.99-1.08) | 0.089 | 1.04 (0.99-1.08) | 0.091 |
| MELD (points) | 1.02 (0.97-1.08) | 0.346 | 1.00 (0.94-1.06) | 0.929 | - | - |
| Albumin (g/L) | 0.94 (0.89-0.98) | 0.007 | 0.95 (0.89-1.00) | 0.057 | 0.95 (0.90-0.99) | **0.032** |
| PRO-C4 (per 100 ng/mL) | 0.98 (0.81-1.17) | 0.793 | 1.13 (0.87-1.46) | 0.359 | - | - |
| C4M (ng/mL) | 0.99 (0.98-1.01) | 0.284 | 0.98 (0.96-1.01) | 0.136 | 0.99 (0.98-1.01) | 0.259 |
| C4ratio | 1.02 (0.91-1.14) | 0.741 | - | - | - | - |
| **C6-biomarkers** | **HR (95%CI)** | **p-value** | **aHR (95%CI)** | **p-value** | **aHR (95%CI)** | **p-value** |
| Age (per year) | 1.01 (0.99-1.04) | 0.220 | 1.02 (0.99-1.04) | 0.189 | - | - |
| HVPG (mmHg) | 1.05 (1.01-1.10) | **0.012** | 1.04 (0.99-1.09) | 0.089 | 1.04 (0.99-1.08) | 0.083 |
| MELD (points) | 1.02 (0.97-1.08) | 0.346 | 1.00 (0.93-1.07) | 0.941 | - | - |
| Albumin (g/L) | 0.94 (0.89-0.98) | **0.007** | 0.95 (0.90-1.00) | 0.072 | 0.95 (0.90-0.99) | **0.035** |
| PRO-C6 (ng/mL) | 1.00 (0.98-1.02) | 0.727 | 1.01 (0.98-1.03) | 0.667 | - | - |
| C6M (ng/mL) | 0.49 (0.06-3.78) | 0.491 | 0.50 (0.05-4.73) | 0.549 | - | - |
| C6ratio | 1.00 (0.99-1.01) | 0.666 | - | - | - | - |
| **ELF score** | **HR (95%CI)** | **p-value** | **aHR (95%CI)** | **p-value** | **aHR (95%CI)** | **p-value** |
| Age (per year) | 1.01 (0.99-1.04) | 0.220 | 1.02 (0.99-1.04) | 0.177 | - | - |
| HVPG (mmHg) | 1.05 (1.01-1.10) | **0.012** | 1.04 (1.00-1.09) | 0.071 | 1.04 (0.99-1.08) | 0.083 |
| MELD (points) | 1.02 (0.97-1.08) | 0.346 | 1.00 (0.94-1.07) | 0.940 | - | - |
| Albumin (g/L) | 0.94 (0.89-0.98) | **0.007** | 0.94 (0.89-1.00) | 0.048 | 0.95 (0.90-0.99) | **0.035** |
| ELF score | 1.15 (0.96-1.38) | 0.129 | 0.95 (0.73-1.23) | 0.692 | - | - |
| **PRO-C18L** | **HR (95%CI)** | **p-value** | **aHR (95%CI)** | **p-value** | **aHR (95%CI)** | **p-value** |
| Age (per year) | 1.01 (0.99-1.04) | 0.220 | 1.02 (0.99-1.04) | 0.184 | - | - |
| HVPG (mmHg) | 1.05 (1.01-1.10) | **0.012** | 1.04 (0.99-1.09) | 0.089 | 1.04 (0.99-1.08) | 0.083 |
| MELD (points) | 1.02 (0.97-1.08) | 0.346 | 1.00 (0.94-1.07) | 0.992 | - | - |
| Albumin (g/L) | 0.94 (0.89-0.98) | **0.007** | 0.95 (0.89-1.00) | 0.057 | 0.95 (0.90-0.99) | **0.035** |
| PRO-C18L (ng/mL) | 1.02 (0.96-1.07) | 0.551 | 1.00 (0.94-1.06) | 0.901 | - | - |

Statistical Analysis: Uni- and multivariable Cox regression analyses of factors associated with first/further hepatic decompensation (according to clinical stage at baseline), development of ACLF and liver-related death in a multistate model (n=48 events). Abbreviations: (aHR) adjusted hazard ratio, (HVPG) Hepatic Venous Pressure Gradient, (MELD) Model for End-Stage Liver Disease, (PRO-C3) Collagen Type III Propeptide, (PRO-C4) Collagen Type IV Propeptide, (PRO-C6) Collagen Type VI Propeptide, (C3M) Collagen Type III Metabolite, (C4M) Collagen Type IV Metabolite, (C6M) Collagen Type VI Metabolite, (C3ratio) Collagen Type III Ratio, (C4ratio) Collagen Type IV Ratio, (C6ratio) Collagen Type VI Ratio, (ELF) enhanced liver fibrosis score, (PRO-C18L) Collagen Type XVIII formation marker.

**Supplementary Table-7. Patient characteristics of patients with cACLD in the derivation and validation cohorts.**

|  | **Derivation cohort (cACLD)**  **N=101** | **Validation cohort (cACLD)**  **N=185** |
| --- | --- | --- |
| Sex (n, %): |  |  |
| F | 36 (35.6%) | 65 (35.1%) |
| M | 65 (64.4%) | 120 (64.9%) |
| Age (years) | 59.2 [51.7;68.2] | 56.9 [49.2;64.6] |
| HVPG (mmHg) | 13.0 [9.00;18.0] | 12.0 [9.00;16.0] |
| Etiology (n, %): |  |  |
| ALD | 23 (22.8%) | 66 (35.7%) |
| Viral | 29 (28.7%) | 37 (20.0%) |
| ALD & Viral | 6 (5.94%) | 9 (4.86%) |
| MASLD | 21 (20.8%) | 33 (17.8%) |
| Cholestatic | 4 (3.96%) | 16 (8.65%) |
| Other | 18 (17.8%) | 24 (13.0%) |
| LSM (kPa)* | 23.4 [16.8;35.3] | 26.6 [16.8;43.8] |
| CTP stage (n, %): |  |  |
| A | 92 (91.1%) | 151 (81.6%) |
| B | 9 (8.91%) | 32 (17.3%) |
| C | 0 (0.00%) | 2 (1.08%) |
| MELD (points) | 9.00 [8.00;12.0] | 9.00 [8.00;13.0] |
| ELF score | 10.7 [9.93-11.5] | 10.9 [10.0;11.9] |
| PRO-C3 (ng/mL) | 14.6 [10.0;21.0] | 28.4 [22.6;39.5] |
| PRO-C6 (ng/mL) | 11.7 [9.37;15.4] | 9.63 [7.50;13.6] |

*LSM available in n=92 (91%) in the derivation cohort with cACLD, and n=182 (98%) in the validation cohort.

Abbreviations: (aHR) adjusted hazard ratio, (HVPG) Hepatic Venous Pressure Gradient, (MELD) Model for End-Stage Liver Disease, (PRO-C3) Collagen Type III Propeptide, (PRO-C6) Collagen Type VI Propeptide, (ELF) enhanced liver fibrosis score, (LSM) liver stiffness measurement.

**Supplementary Table-8. Cox regression models for the prediction of disease progression in patients with compensated ACLD (cACLD): validation cohort.**

| **cACLD**  **PRO-C3** | **Univariable** | | **Multivariable (first model)** | | **Multivariable (last model)** | |
| --- | --- | --- | --- | --- | --- | --- |
|  | **HR (95%CI)** | **p-value** | **aHR (95%CI)** | **p-value** | **aHR (95%CI)** | **p-value** |
| Age (per year) | 0.99 (0.97-1.02) | 0.877 | 0.99 (0.96-1.04) | 0.928 |  |  |
| HVPG (mmHg) | 1.18 (1.12-1.24) | **<0.001** | 1.16 (1.08-1-24) | **<0.001** | 1.16 (1.10-1.23) | **<0.001** |
| MELD (points) | 1.08 (1.04-1.13) | **<0.001** | 1.00 (0.88-1.14) | 0.967 |  |  |
| Albumin (g/L) | 0.89 (0.83-0.95) | **<0.001** | 0.93 (0.85-1.01) | 0.093 | 0.92 (0.86-0.98) | **0.013** |
| PRO-C3 (ng/mL) | 1.04 (1.01-1.06) | **0.004** | 1.01 (0.98-1.05) | 0.374 |  |  |
| **PRO-C6** | **HR (95%CI)** | **p-value** | **aHR (95%CI)** | **p-value** | **aHR (95%CI)** | **p-value** |
| Age (per year) | 0.99 (0.97-1.02) | 0.877 | 0.99 (0.96-1.04) | 0.983 |  |  |
| HVPG (mmHg) | 1.18 (1.12-1.24) | **<0.001** | 1.16 (1.08-1-24) | **<0.001** | 1.16 (1.10-1.23) | **<0.001** |
| MELD (points) | 1.08 (1.04-1.13) | **<0.001** | 0.99 (0.87-1.15) | 0.985 |  |  |
| Albumin (g/L) | 0.89 (0.83-0.95) | **<0.001** | 0.93 (0.85-1.00) | 0.063 | 0.92 (0.86-0.98) | **0.013** |
| PRO-C6 (ng/mL) | 1.02 (1.00-1.05) | 0.094 | 1.02 (0.99-1.05) | 0.266 |  |  |
| **ELF score** | **HR (95%CI)** | **p-value** | **aHR (95%CI)** | **p-value** | **aHR (95%CI)** | **p-value** |
| Age (per year) | 0.99 (0.97-1.02) | 0.877 | 1.00 (0.96-1.04) | 0.846 |  |  |
| HVPG (mmHg) | 1.18 (1.12-1.24) | **<0.001** | 1.15 (1.07-1.23) | **<0.001** | 1.16 (1.10-1.23) | **<0.001** |
| MELD (points) | 1.08 (1.04-1.13) | **<0.001** | 1.00 (0.87-1.15) | 0.988 |  |  |
| Albumin (g/L) | 0.89 (0.83-0.95) | **<0.001** | 0.94 (0.85-1.03) | 0.200 | 0.92 (0.86-0.98) | **0.013** |
| ELF score | 1.59 (1.28-1.96) | **<0.001** | 1.14 (0.80-1.63) | 0.470 |  |  |

Statistical Analysis: Uni- and multivariable Cox regression analysis of factors associated with first hepatic decompensation. Abbreviations: (aHR) adjusted hazard ratio, (HVPG) Hepatic Venous Pressure Gradient, (MELD) Model for End-Stage Liver Disease, (PRO-C3) Collagen Type III Propeptide, (PRO-C6) Collagen Type VI Propeptide, (ELF) enhanced liver fibrosis score.

**Supplementary Table-9. Comparison of studies on ECM remodeling biomarkers in chronic liver disease.**

| **PMID** | **Selection** | **Size** | **Biomarkers** | **Main result/focus** | **Comment** |
| --- | --- | --- | --- | --- | --- |
| Present manuscript  (Simbrunner, Villesen et al) | ACLD (cACLD and stable dACLD; i.e., AD/ACLF EXCLUDED) | 232 ACLD | ELF  PRO-C3  PRO-C4  PRO-C6  PRO-C18L  C3M  C4M  C6M | ELF and fibrogenesis biomarkers increase between cACLD and dACLD (sub-stages) and correlate with HVPG  (Independent) predictive value of ECM biomarkers restricted to cACLD  Adjusted Cox models suggest that increased ECM degradation markers indicate REDUCED risk of disease progression | Large cohort representative of contemporary “outpatients” with c/dACLD  Prospective characterization including HVPG and follow-up  Prognostic value of ECM biomarkers assessed |
| 35822301  (Simbrunner et al) | ACLD (cACLD and stable dACLD i.e., AD/ACLF EXCLUDED) | 215 ACLD | **ELF**  **PRO-C3**  **PRO-C6**  **C3M** | ECM Biomarkers linked to systemic inflammation  Relationship between ECM markers and HVPG shown to justify statistical adjustments in regression analysis | Many biomarkers not measured **& no assessment of prognostic value**  Pathophysiology-focused study on systemic inflammation and ECM remodelling |
| 34805815  (Kerbert et al.) | dACLD (**AD and ACLF**) | 283 AD-ACLF  64 stable ACLD  30 controls | **PRO-C3,**  **PRO-C4**,  PRO-C5,  **PRO-C6**,  PRO-C8  **C4M**  **C6M** | PRO-C3 and PRO-C6 increased in AD/ACLF and correlate with liver dysfunction & inflammation  PRO-C6 correlates with 28- and 90-day mortality in ACLF | **Focus on AD/ACLF (i.e., patients excluded from present study)**  No information on prognostic value in cACLD and stable dACLD **(i.e., patients included in our study)**  No information on HVPG |
| 29404528  (Praktiknjo et al.) | dACLD (**AD**) | 110 dACLD (**AD**) | **PRO-C3**  **C3M** | PRO-C3 correlates with MELD and other disease severity scores in AD  PRO-C3/C3M ratio correlates with risk for ACLF and mortality | **Distinct subgroup of dACLD (AD – TIPS)**  Only 2 biomarkers  No multivariate analyses for prognostic assessment |
| 24099470  (Leeming et al.) | ACLD (stages unknown) | 94 ACLD  20 controls | **PRO-C3**  P4NP7S  BGM (biglycan),  ELM (elastin)  C1M  **C3M**  **C4M**  C5M  **C6M** | ECM markers correlate with HVPG  Models for prediction of HVPG ≥10 mmHg | Partially overlapping biomarker panel  Small cohort  **Relationship with disease stages and prognosis not assessed – dACLD not within the target population for HVPG ≥10 mmHg detection**  Relationship between biomarkers and HVPG consistent with results in cACLD subgroup of the present study |
| 25265505  (Jansen et al.) | Non-ACLD & ACLD | 58 (HIV-HCV, predominantly non-ACLD) | **PRO-C3**  **C4M**  C5M | PRO-C3 correlates with HVPG (r = 0.354)  C4M and C5M increase in patients with portal hypertension (HVPG ≥6 mmHg) | **HVPG <6mmHg in most patients, i.e., non-ACLD (i.e., patients excluded from the present study)**  Only 3 biomarkers; 2 overlapping with present study  Small cohort  Clinical utility of detecting HVPG ≥6 mmHg unclear  **No outcomes evaluated**  HVPG only available in 43 / 58 patients |
| 26406331  (Nielsen, M.J., et al.) | Non-ACLD & cACLD | 321 ≤F2 (non-ACLD)  80 F3/F4 (cACLD) | **PRO-C3**  P4NP7S  C1M  **C3M**  **C4M**  **C6M** | PRO-C3 increased in ≥F2 compared to F0/F1  C3M, C4M, C6M increased in ≥F3 compared to F0-2 | **Mostly non-ACLD patients,** only partially overlapping biomarker panel  No dACLD patients  **No information on HVPG**  **No clinical outcome assessment** |
| 30120271  (Luo et al.) | Non-ACLD & cACLD | 164 (MASLD, mostly non-ACLD)  41 paired biopsies | **PRO-C3**  PRO-C5  **PRO-C6**  P4NP7S  **C3M**  **C4M** | PRO-C3 and PRO-C6 elevated in F3/F4 compared to ≤F2  Paired biopsy: PRO-C3 increase associated with worsening of fibrosis | **Mostly non-ACLD patients & no dACLD patients**  Only partially overlapping biomarker panel  **No information on HVPG**  **No clinical outcome assessment** |

Legend: Biomarkers **overlapping** with the present study and **important between-study differences** are highlighted **in bold**. Abbreviations: (c/dACLD) compensated/decompensated advanced chronic liver disease; (AD) acute decompensation; (ACLF) acute-on-chronic liver failure; (ALD) alcohol-related liver disease; (ELF) enhanced liver fibrosis score; (TIMP1) tissue inhibitor of metalloproteinases 1; (HA) hyaluronic acid; (P3NP) Procollagen 3 N-terminal peptide); (PRO-C3/C4/C5/C6/C18L) Collagen Type III/IV/V/VI/VIII/XVIII Propeptide; (C1M/C3M/C4M//C5M/C6M) Collagen Type I/III/IV/V/VI metabolite; (P4NP 7S) N-terminal pro-peptide of type IV collagen 7S domain; (BGM) biglycan; (ELM) elastin; (MELD) model for end-stage liver disease; (TIPS) transjugular intrahepatic portosystemic shunt; (HIV) human immunodeficiency virus; (HCV) hepatitis C virus; (HVPG) hepatic venous pressure gradient.

# References (used in the supplementary material)

1. de Franchis, R., et al., *Baveno VII - Renewing consensus in portal hypertension.* J Hepatol, 2022. **76**(4): p. 959-974.

2. Heinze, G. and D. Dunkler, *Five myths about variable selection.* Transpl Int, 2017. **30**(1): p. 6-10.
